# A Microenvironment‐Adaptive Scaffold Rejuvenates Peripheral Nerve Regeneration During Aging Via Dual Modulation of Ferroptotic Stimuli in Schwann Cells

**DOI:** 10.1002/advs.77351

**Published:** 2026-08-25

**Authors:** Ning Zhan, Lixin Tian, Lianjie Peng, Jingyu Zhang, Lining Lin, Xiaoting Zhang, Yaxian Luo, Zhichao Liu, Xinhua Yao, Mouyuan Sun, Yong He, Mengfei Yu, Huiming Wang

**Affiliations:** ^1^ Stomatology Hospital School of Stomatology Zhejiang University School of Medicine Zhejiang Provincial Clinical Research Center for Oral Diseases Key Laboratory of Oral Biomedical Research of Zhejiang Province Cancer Center of Zhejiang University Engineering Research Center of Oral Biomaterials and Devices of Zhejiang Province Hangzhou China; ^2^ Department of Plastic Surgery The First Affiliated Hospital School of Medicine Zhejiang University Hangzhou China; ^3^ State Key Laboratory of Fluid Power and Mechatronic Systems School of Mechanical Engineering Zhejiang Key Laboratory of Additive Manufacturing Technology and Equipment School of Mechanical Engineering Zhejiang University Hangzhou China

## Abstract

Repairing peripheral nerve injury remains a clinical challenge due to its limited regenerative capacity and the situation is further compounded with age. In this study, we identified the dysregulated repair program of Schwann cells (SCs) as a key feature of the aging‐dependent impairment of nerve regeneration, mediated by dual influence of ferroptosis. The ferroptosis‐stressor‐rich microenvironment inhibits SC function in regenerating nerves. Moreover, the persistent intracellular ferroptosis stressor accumulation could accentuate senescence‐associated secretory phenotype (SASP) expression in SCs, culminating in the disruption of redifferentiation trajectory and repair programs of SCs. Herein, a composite nerve conduit integrating microenvironment‐responsive Ga‐PTA nanoparticles and maxillofacial‐derived mesenchymal stem cells (Mmscs) is developed by melt electrospinning writing (MEW). Mechanistically, Ga‐PTA nanoparticles competitively inhibited excessive ferroptosis to establish a pro‐regenerative niche. Meanwhile, Mmscs was induced to differentiate into SC‐like cells which bypassed the redifferentiation impairment of resident SASP‐expressing SCs, offering promising therapeutic strategies for peripheral nerve and other tissue injuries during aging.

## Graphical abstract

(i) Aging impairs peripheral nerve regeneration via its dual ferroptosis‐mediated pathologies in senescent schwann cells. (ii) The composite nerve conduit is a symbiosis niche integrating microenvironment‐responsive nanoparticles (Ga‐PTAs) and maxillofacial‐derived mesenchymal stem cells (Mmscs). (iii) Under the induction of high ferroptosis levels in nerve stump, Ga‐PTA could competitively inhibit excessive ferroptosis and induce Mmscs to diffrentiate into schwann cell‐like cells.

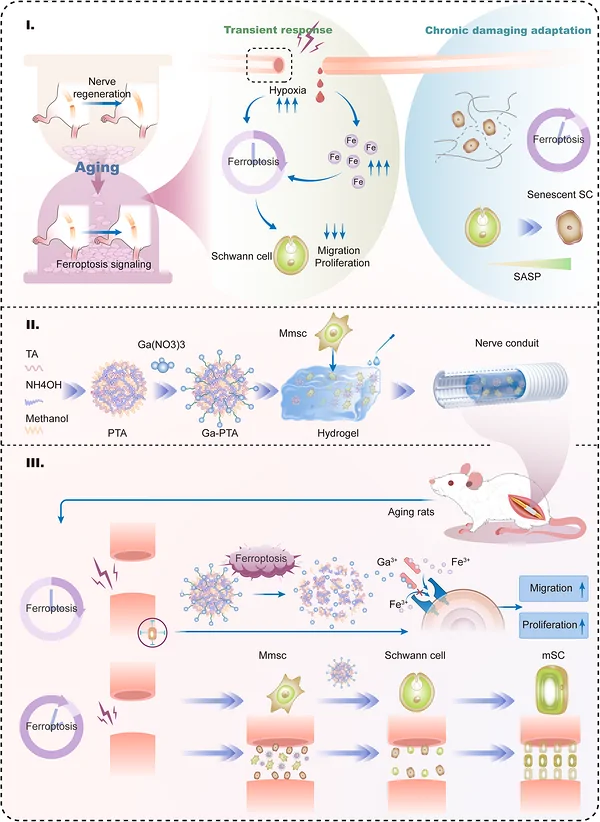

## 1 Introduction

Nowadays, with the continuing increase of the elderly segment of the population, motor impairment associated with heterogeneous etiologies has become a critical concern, which could cause injuries in muscles, nerves and bones [1]. Among them, peripheral nerve injuries (PNI) are critical as they may result in alterations in neuro‐osseous interactions and denervation of distal musculature, leading to severe sensory and motor dysfunctions and creating significant clinical and economic burdens [2, 3, 4, 5]. Although peripheral nerves possess a degree of spontaneous repair capacity post‐injury, the regeneration of axons and restoration of nerve function diminish with age and can be adversely affected by various interfering factors, which requires surgical treatment [6, 7]. The current clinical gold standard to replace the lost nerve tissue is autologous nerve grafting, as it provides appropriate cellular and extracellular matrix support for nerve regeneration. However, this method still faces a series of inevitable issues, including insufficient donor nerves, mismatched nerve fibers, donor nerve impairment, and time‐consuming secondary surgeries [8, 9]. Other surgical treatments such as suture and allogeneic nerve grafting could promote nerve reconstruction to some extent; yet, there remain obvious problems such as excessive suture tension and immune rejection [10, 11]. As an alternative, artificial nerve repair conduits have been utilized to create a suitable microenvironment, minimizing adverse factors that interfere with nerve repair and guiding axonal regeneration [12, 13]. However, existing nerve conduit designs focus solely on factors directly promoting nerve regeneration but neglect the impact of optimizing the regenerative microenvironment in aging individuals, resulting in limited effectiveness in promoting nerve regeneration in aging individuals [14, 15]. Hence, there is an urgent need for in‐depth investigations into the regenerative mechanisms underlying PNI in aged individuals, which will facilitate the development of precise and efficient therapeutic strategies to promote neural repair.

Previous studies have identified age as a significant interfering factor affecting nerve regeneration in patients with PNI and experimental animal models. The decline in aging‐dependent peripheral nerve regeneration capabilities is not due to a decreased capacity for axonal extension in neurons but rather stems from insufficient repair status of Schwann cells [16]. Senescent Schwann cells in the nerve injury site exhibit diminished proliferation capacity and secrete substances that inhibit axonal growth in a state of chronic inflammation, which interferes with normal Schwann cell repair programs. The disturbance of Schwann cell repair programs adversely affecting the extension of Schwann cell cord and remyelination, ultimately limiting axonal extension [7]. Although abnormalities in Schwann cells have been established as a primary cause of aging‐related limitations on peripheral nerve regeneration, the underlying mechanisms that cause these repair program anomalies remain unclear.

Ferroptosis is a novel form of iron‐dependent programmed cell death driven by the accumulation of lethal lipid peroxidation and reactive oxygen species (ROS). The biological characteristics of ferroptosis include ROS accumulation, iron overload, rupture of the outer mitochondrial membrane, and lack of chromatin condensation [17, 18]. The local hemorrhage and hypoxia following traumatic PNI can exacerbate iron loading and the accumulation of free radicals that initiate ferroptosis, which is detrimental to nerve regeneration. In aging individuals, the weakening of the intracellular antioxidant system further exacerbates ferroptosis of peripheral nerves post‐injury [19, 20]. Our research revealed a significant correlation between the insufficient repair status of senescent Schwann cells and ferroptosis levels. Based on previous studies, we described that response to the ferroptosis‐related stressor exposure state in Schwann cells at the nerve injury site in aging individuals could be categorized into transient responses and chronic damaging adaptations [21]. The former primarily causes reversible inhibition of the proliferation and migration functions of local cells, while chronic damaging adaptations leads to an accumulation of adverse factors within Schwann cells, ultimately resulting in irreversible alterations in the repair program of Schwann cells.

Accordingly, to precisely achieve promotion of the peripheral nerve repair process in aging individuals, the following two primary issues must be addressed: first, effectively reducing the excessive ferroptosis levels at the injury site in aging individuals; second, restarting the repair program of Schwann cells and promoting peripheral nerve regeneration. Herein, we designed a novel composite therapeutic nerve scaffold to address the two main factors aforementioned. We first used a modified Stöber method to prepare a gallium ion‐poly (tannic acid) composite nanoparticles (Ga‐PTAs) as a carrier for gallium ion (Ga^3+^) donor. By responsive releasing Ga^3+^ in a ferroptosis microenvironment, these nanoparticles can competitively reduce the accumulation of intracellular free ferrous ions to inhibit ferroptosis. Simultaneously, we employed melt electrospinning writing (MEW) technology to fabricate a conduit with aligned fibers, which could facilitate cell directionality and adhesion. Then the Ga‐PTA nanoparticles and maxillofacial‐derived mesenchymal stem cells (Mmscs) were loaded into the nerve guiding conduit together with a carrier (GelMA‐Matrigel composite hydrogel). The release of Ga^3+^ could reduce ferroptosis levels, induce the differentiation of Mmscs into Schwann cells at the injury site and improve the repair status of Schwann cells, ultimately promoting precise nerve repair in aging organisms.

## 2 Results

### 2.1 Ferroptosis Signaling Activated in Senescent Schwann Cells Attenuates Peripheral Nerve Regeneration in Aging Rats

**FIGURE 1:**
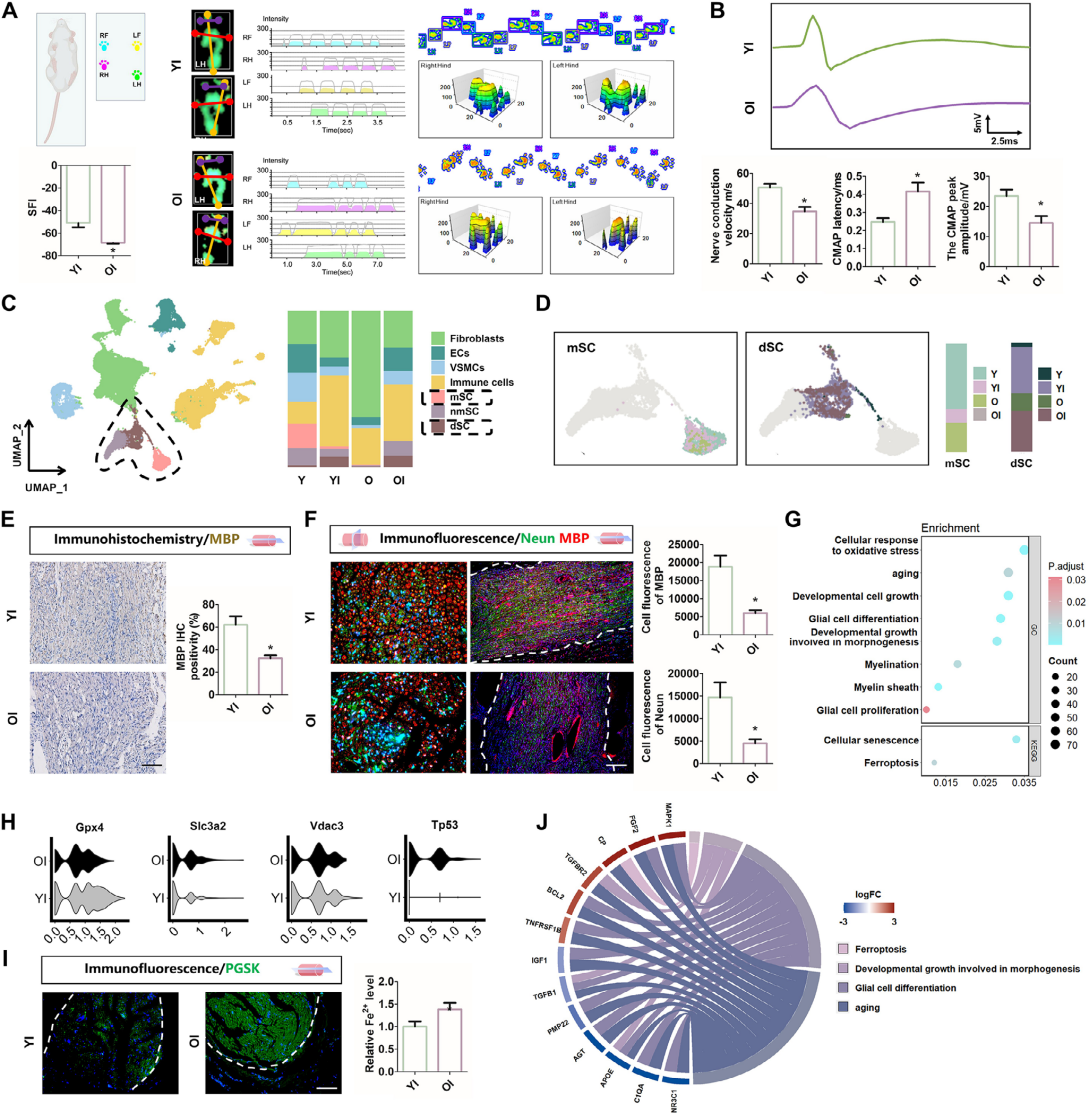
The aging rats harbors the impairment of peripheral nerve regeneration and the activation of ferroptosis signaling. (A) Functional recovery analysis via catwalk gait at 16 weeks post injury. The sciatic nerve indexes (SFI) is also evaluated. RF, right front limb; RH, right hind limb; LF, left front limb; LH, left hind limb. (B) Electrophysiological assessment of CMAP for different aspects (the NCV, the latency period of CMAPs, and the peak amplitude of CMAPs). (C) UMAP representation of major cell types identified by scRNA‐seq. Bar graph of relative cell proportions. (D) UMAP of mSC (left) and dSC (right) in Y, YI, O and OI. Bar graph of relative cell proportions by groups. (E) The images of immunohistochemistry staining of MBP. n = 3 per group. Quantitative analysis of intensity is also shown. Scale bar = 50 µm. (F) Immunofluorescence images for the localization and the expression of Neun (green), MBP (red) and DAPI (blue). n = 3 per group. Quantitative analysis of intensity is also shown. Scale bar = 50 µm. (G) Dot plots of GO enrichments and KEGG pathways of Schwann cells in OI. (H) Violin plots of ferroptosis‐related genes in Schwann cells and relative expression per group. X‐axis represents normalized average expression. (I) Immunofluorescence images for the localization and the expression of PGSK (green) and DAPI (blue). n = 3 per group. Quantitative analysis of intensity is also shown. Scale bar = 50 µm. (J) Chord diagrams of ferroptosis, developmental growth involved in morphogenesis, glial cell differentiation and aging enrichments. *p < 0.05, **p < 0.01, ***p < 0.001. Y, young control group; YI, young nerve injury group; O, aging control group; OI, aging nerve injury group.

To investigate how aging affects the regeneration process after PNI, we induced sciatic nerve transection injury models in young (8‐week‐old) and aging (21‐month‐old) rats and treated the models with autologous nerve anastomosis. 16 weeks after the surgery, hematoxylin‐eosin (HE) and toluidine blue (TB) staining were used to assess the histological recovery of sciatic nerve (Extended Data Figure S1A,B). Upon aging, we observed a decrease of nerve fiber uniformity, myelinated axon density and myelin sheath thickness in the regenerated sciatic nerve. The neuromotor function was assessed by Catwalk gait automatic analyze and the Sciatic Nerve Functional Index (SFI) was calculated to describe toe spread. Compared to young control, the aging rat had a reduction in contact intensity and SFI of the hind limb on the surgical side (Figure 1A). We further compared the nerve conductivity recovery between aging and young groups via neuroelectrophysiology. The result showed that the amplitude of compound muscle action potential (CMAP) and nerve conduction velocity (NCV) were decreased in the aging group (Figure 1B).

Considering the cellular heterogeneity in peripheral nerve, we employed single‐cell RNA sequencing (scRNA‐seq) to explore the specific mechanism occurring with age within the regenerated nerve. We isolated 44 843 high‐quality cells from the peripheral nerve of the young and aging sciatic nerve chronic constriction injury (CCI) models for analysis after quality filtering. The data of the injured and normal sciatic nerve of the young control groups were obtained from published data sets (GSE216665) [22] (Extended Data Figure S1C–E). The cells in the integrated scRNA seq datasets were classified into 7 major cell compartments by the UMAP algorithm comprised of vascular smooth muscle cells (VSMCs), endothelial cells (ECs), fibroblasts, immune cells, dedifferentiated Schwann cells (dSCs), myelinating Schwann cells (mSCs), and non‐myelinating Schwann cells (nmSCs) (Figure 1C). Analysis of the transcriptomic signatures for the major cell types documented differential expression of classical markers for the 7 major cell compartments, which were generally conserved across peripheral nerves (Extended Data Figure S1F,I). Representation of these 7 main cell types was different between aging and young groups, as the aging sciatic nerves contained significantly constrictive mSC compartments, as well as expanded dSC compartments (Extended Data Figure S1G,H). Myelinating Schwann cells (mSCs) and non‐myelinating Schwann cells (nmSCs) represent the two predominant mature Schwann cell types in the peripheral nervous system. Specifically, mSCs wrap individual axons with a diameter >1 µm to form a myelin sheath essential for rapid saltatory impulse propagation, while nmSCs are critical in maintaining the integrity and functionality of unmyelinated axons [23]. After nerve damage, SCs could response to neurotrauma and transition to a dedifferentiated state, called dedifferentiated Schwann cells (dSCs). Such Schwann cells initiate regeneration by recruiting macrophages to the lesion site [24, 25]. Focusing on the major cell types that changed in elderly rats, we reclustered the cells identified as mSCs and dSCs to obtain a more refined view of these populations. And the compartment of mSC showed further reduced proportions in the aging sciatic nerve injury group (Figure 1D). We performed further immunohistochemistry to examine the expression of the mSC marker MBP in the regenerated nerves. The images of longitudinal section showed that the MBP‐positive expressed areas in the young sciatic nerve transection injury group were significantly larger than the aging group (Figure 1E). Likewise, Immunofluorescence staining of transected and longitudinal section showed that the fluorescence distributions of Neun (labeled neuronal cells) and MBP in the young injury group were more widely and continuously distributed than that in the corresponding aging group (Figure 1F). Therefore, we surmised that the difference in the sciatic nerve repair effect between young and aged rats may primarily due to the obstruction in the imbalance of mSCs and dSCs in the regenerating nerves of aged rats.

We next investigated the mechanism of these difficulties in nerve regeneration in aging. The GO enrichment analysis and KEGG pathway analysis results indicated that aging influenced pathways related to myelination and iron metabolism in Schwann cells (Extended Data Figure S1J). Likewise, Schwann cells in regenerative nerves from aged rats accentuate alterations in remyelination‐related and ferroptosis‐related enrichments compared with the corresponding young group (Figure 1G). Consistently, ferroptosis‐related gene p53 tumor suppressor protein (Tp53) exhibited marked upregulation while glutathione peroxidase 4 (Gpx4) and voltage dependent anion channel 3 (Vdac3) were downregulated to unleash ferroptosis in Schwann cells of aging rats with nerve injury (Figure 1H, Extended Data Figure S1K). Pro‐ferroptosis signaling in aged nerves was further monitored by staining of PGSK dye (a probe of intracellular ferrous iron). The fluorescence intensities of PGSK were dramatically upregulated in injured nerves (Figure 1I). The chord diagram depicted the potential pleiotropic genes shared among ferroptosis, aging, developmental growth involved in morphogenesis and glial cell differentiation pathways, implying a relevance among these pathways (Figure 1J). These observations demonstrated that ferroptosis signaling is activated in senescent Schwann cells and may attenuate peripheral nerve regeneration in aging rats.

### 2.2 Ferroptosis‐Responsive Self‐Disintegrating Nanoparticles Ameliorates the Function of the Senescent Schwann Cells

**FIGURE 2:**
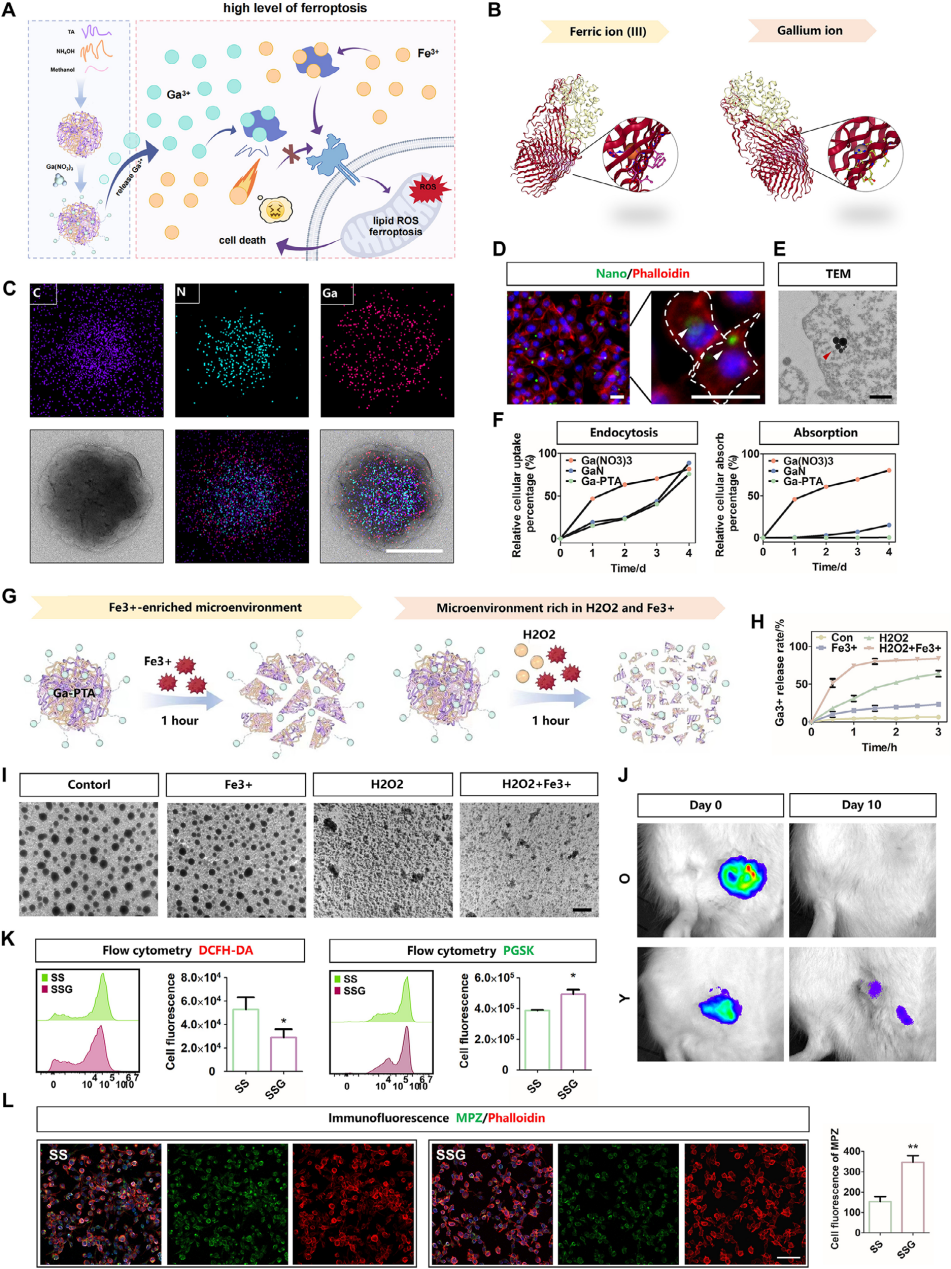
Ferroptosis‐responsive self‐disintegrating nanoparticles ameliorates the function of the senescent Schwann cells. (A) Schematic illustration of the synthesis and disintegration of Ga‐PTA nanoparticles. (B) 3D‐molecular docking between gallium ion/ferric ion and transferrin. (C) TEM image and elemental mapping for Ga‐PTA nanoparticles. Scale bar = 50 nm. (D) Immunofluorescence images of cellular uptake of the Ga‐PTA nanoparticles (green) in RSC96 cells. Scale bar = 50 µm. (E) TEM image of cellular uptake of the Ga‐PTA nanoparticles in RSC96 cells. Scale bar = 500 nm. (F) Intracellular uptake (left) and absorption (right) metal mass of gallium normalized to cell number as quantified by ICP‐MS. (G) The schematic diagram of the disintegration of Ga‐PTA nanoparticles in a hydrogen peroxide‐Fe^3+^ solution. (H) Gallium ions release profiles from Ga‐PTA NPs in a hydrogen peroxide‐Fe^3+^ solution. (I) The TEM images of Ga‐PTA incubated with hydrogen peroxide‐Fe^3+^ solution. Scale bar = 200 nm. (J) The fluorescence images of the biodistribution and elimination of Ga‐PTA nanoparticles in vivo. (K) The intracellular ROS and ferrous ion levels of senescent RSC96 cell models after Ga‐PTA intervention evaluated by flow cytometry. (L) Immunofluorescence staining for MPZ (green) and actin (red) in senescent RSC96 cells with Ga‐PTA treatment, and quantitative analysis of MPZ fluorescence intensity. Scale bar = 100 µm. *p < 0.05, **p < 0.01, ***p < 0.001. Y, young control group; O, aging control group; SS, senescent Schwann cell models; SSG, Ga‐PTA‐intervention senescent Schwann cell models.

Gallium, a group IIIa element, has a nearly ionic radius with iron, while Ga^3+^ cannot transfer to a divalent state to participate in redox reactions [26]. Through molecular docking, we found that Ga^3+^ could competitively bind to the Fe^3+^‐binding site on transferrin (a key protein involved in iron metabolism and ferroptosis), ultimately preventing the entry of Fe^3+^ into cells (Figure 2B). Therefore, we hypothesize that Ga^3+^ may suppress excessive ferroptosis in the nerve defect of aged rats. However, the instability, poor bioavailability and dose‐dependent toxicity of current gallium‐based compounds limit their clinical application. To address these issues, we synthesized Ga‐PTA nanoparticles via phenol‐formaldehyde condensation, coordination reaction and hydrothermal treatment (Figure 2A). PTA nanoparticles, as control, were prepared according to the same procedure except coordination reaction. The morphology of Ga‐PTA nanoparticles were observed by SEM and the result was shown that Ga‐PTA nanoparticles were uniform spheres at an average size of 29.8 ± 24.2 nm (Extended Data Figure S2A). The zeta potential was −33.7 mV for hydrated PTA nanoparticles and −31.2 mV for hydrated Ga‐PTA nanoparticles evaluated by dynamic light scattering (DLS). The absolute values of both zeta potentials are larger than 30 mV, suggesting PTA and Ga‐PTA nanoparticles theoretically possess colloidal stability (Extended Data Figure S2B). The pattern of X‐ray powder diffraction (XRD) confirmed that compared to PTA, Ga‐PTA nanoparticles successfully introduced Gallium through coordination reaction (Extended Data Figure S2C). Meanwhile, the map of energy‐dispersive X‐ray spectroscopy (EDX) demonstrated that elements (including carbon, nitrogen, and gallium) distributed homogeneously throughout the entire particles (Figure 2C).

Growing evidence has shown that nanoparticles exert their function by entering the cells. Confocal imaging revealed that nanoparticle (Alexa 594 fluorescent label) uptake could be observed in RSC96 cells after 48 h of co‐culture (Figure 2D). Consistently, TEM image of the co‐culture system (for 48 h) showed that Ga‐PTA nanoparticles entered cells and were located in the cytoplasm (Figure 2E). We compared the uptake and degradation of Ga‐PTA nanoparticles by cells with the direct addition of Ga^3+^ or gallium nitride (conventional gallium nanoparticles) using ICP‐MS (inductively coupled plasma‐mass spectrometry) after co‐culture from 1 to 4 days. And the result indicate that Ga‐PTA nanoparticles exhibit superior endocytic efficiency and Ga^3+^ sustained release properties (Figure 2F).

We attempt to test the controlled Ga^3+^ release capability under inflammatory microenvironment through using 2 M H_2_O_2_ solution, 100mM FeCl_3_ solution or PBS at pH = 6 with the addition of HCl (Figure 2G). TEM imaging proved that Ga‐PTA nanoparticles partly disintegrated after being stimulated by H_2_O_2_ for 1 h. After co‐incubation with Fe^3+^ and H_2_O_2_ for 1 h, Ga‐PTA nanoparticles became fragmented and lost spherical particle structure (Figure 2I). Absorbance measurements of the supernatant revealed that Ga‐PTA nanoparticles disintegrated more rapidly and reached the peak of Ga^3+^ release in approximately 1 h after incubating with Fe^3+^ and H_2_O_2_ solution than that incubating with H_2_O_2_ solution (Figure 2H). These results demonstrated that the Ga^3+^ content depended closely on the stimulation of ferroptosis microenvironment (manifested as ROS accumulation and elevated Fe^2+^/Fe^3+^ levels) and exhibited time‐dependent release behavior. To further validate whether these nanoparticles exhibit similar characteristics in the internal environment, we labeled Ga‐PTA nanoparticles with the Cy7 dye and administered them to the injury sites of aged or young rats for 10 days. Consistent with the simulation environment in vitro, these nanoparticles were decomposed more rapidly in aged rats which had an elevated levels of ferroptosis (Figure 2J). These results indicated the ferroptosis‐response self‐disintegrating characteristic of Ga‐PTA nanoparticles, which was desired upon the application at the defect of aged nerves for controlled release of Ga^3+^.

To investigate the effects of these nanoparticles on senescent Schwann cells, we treated RSC96 cells with H_2_O_2_ to construct a premature cell aging model. We co‐cultured the prepared nanoparticles with H_2_O_2_‐induced RSC96 cell senescence models in vitro for 48 h. The results of live‐death staining demonstrated that the cell viability was decreased following treatment with H_2_O_2_ and nanoparticle addition exhibited no significant toxicity to the cells (Extended Data Figure S2D). CCK‐8 assays demonstrated that the proliferation activity of the senescent Schwann cell models after Ga‐PTA intervention could restore closely to the young control level (Extended Data Figure S2E). And wound healing experiments showed that the addition of these nanoparticles promoted the originally weak migration of senescent Schwann cells (Extended Data Figure S2F). We further compared the level of ferroptosis signaling between the senescent Schwann cell models before and after intervention. Flow cytometry analysis revealed that these nanoparticles reduced intracellular ROS and ferrous ion levels (Figure 2K). Additionally, coculturing with Ga‐PTA nanoparticles also enhanced the synthesis of MPZ (myelin protein zero) and NGFR (nerve growth factor receptor) in the senescent Schwann cell model (Figure 2L, Extended Data Figure S2G). All these data above are consistent with our design that Ga‐PTA nanoparticles disintegrate in the high‐ferroptosis‐level microenvironment and affect the function of the senescent Schwann cell models by reducing the level of ferroptosis signaling.

### 2.3 Accumulation of Chronic Damaging Adaptations of Ferroptosis Irreversibly Blocks the Subtype Conversion of Senescent Schwann Cells

**FIGURE 3:**
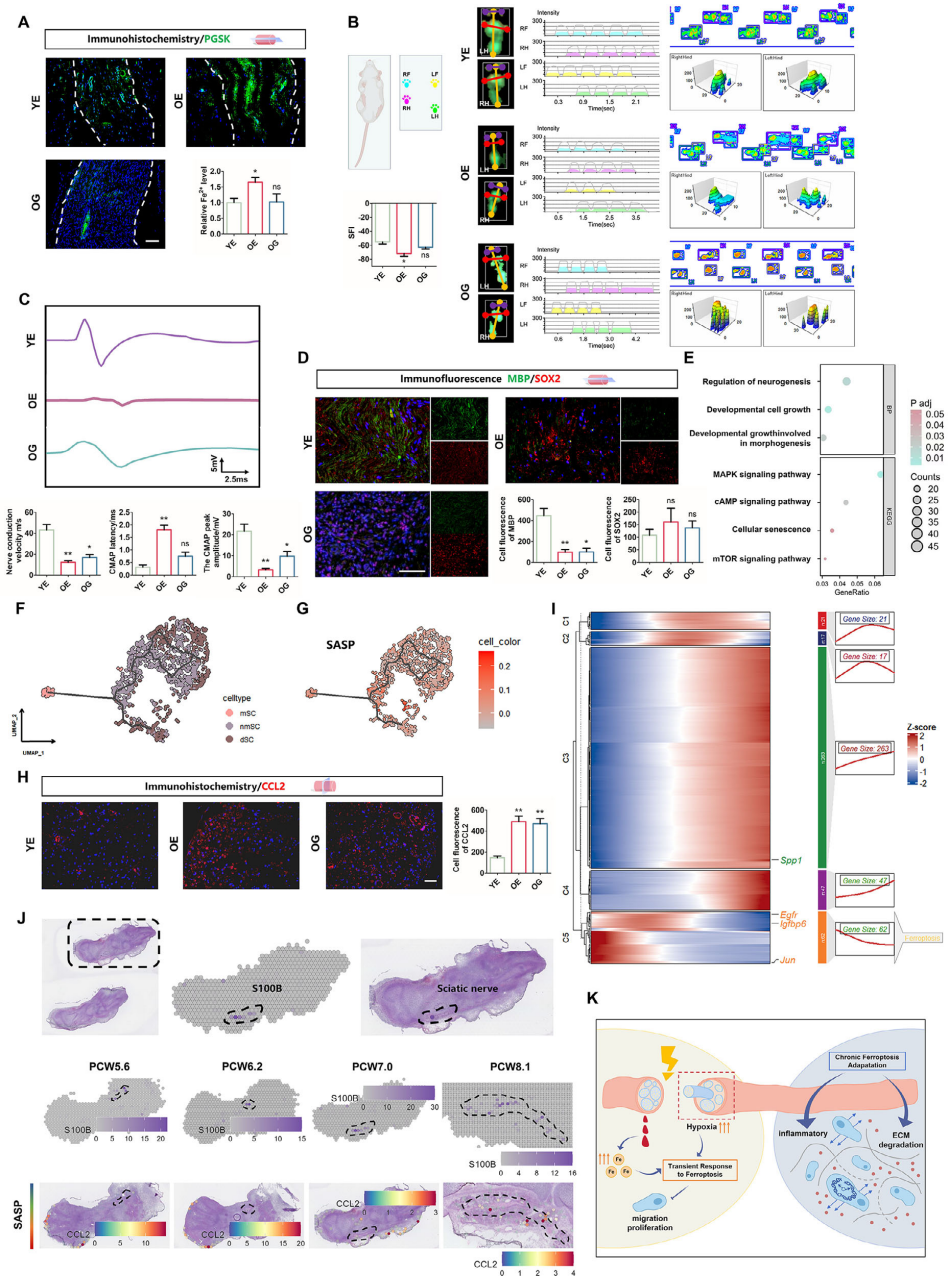
Accumulation of chronic damaging adaptations of ferroptosis irreversibly blocks the subtype conversion of senescent Schwann cells. (A) Immunofluorescence staining for ferrous ion in the regenerated nerves from aging rats after Ga‐PTA intervention. Scale bar = 50 µm. (B) Functional recovery analysis via catwalk gait. The SFI is also evaluated. (C) Electrophysiological assessment of CMAP for different aspects (the NCV, the latency period of CMAPs, and the peak amplitude of CMAPs). (D) The immunofluorescence images of MBP (green), SOX2 (red) and DAPI (blue) in the aging regenerated nerves after Ga‐PTA intervention. Scale bar = 50 µm. (E) Dot plots of GO enrichments and KEGG pathways of Schwann cells in OI. (F) Pseudotime trajectory analysis showing cells ordered by cell type in the aging group. (G) The level of SASP is overlaid on the pseudotime trajectory map. (H) The immunofluorescence images of CCL2 (red) and DAPI (blue) in the aging regenerated nerves after Ga‐PTA intervention. Scale bar = 50 µm. (I) Heatmap showing the differential gene expression patterns of cells with different cell destinies. SASP genes and ferroptosis signaling pathway are annotated on the right side of the heatmap. (J) Spatially resolved heatmaps across tissue sections from the human hindlimbs. Scheme to identify the marker gene of sciatic nerve. Spatial mapping of gene expression for the key node genes in the SASP signaling network in different stage of the developing limb. (K) Dual ferroptosis‐mediated pathologies in senescent Schwann cells during the age‐dependent nerve regeneration decline. *p < 0.05, **p < 0.01, ***p < 0.001. YE, young hollow conduit group; OE, aging hollow conduit group; OG, aging Ga‐PTA‐intervention group.

We next established a model of sciatic nerve injury in aged rats and connected both nerve stumps with either empty nerve conduits or nerve conduits containing Ga‐PTA nanoparticles post injury. In the 16th postoperative week, we found that the regenerated nerves within conduits from aged rats exhibited an elevated level of Ferrous ion and a diminished expression of GPX4 (glutathione peroxidase 4). The Ga‐PTA intervention significantly reduced the level of Ferrous ion and enhanced the GPX4 expression in the injured site (Figure 3A, Extended Data Figure S3A). Still, the nerve regeneration assessment and nerve conduction recovery of the aged rats treated with Ga‐PTA nanoparticles were inferior to those observed in the empty nerve conduit grafting young group (Extended Data Figure S3B, Figure 3B,C). We further examine the proportion of different subtypes of Schwann cells in the regenerated nerves. The Immunofluorescence images of longitudinal section showed that the MBP‐positive expressed areas in the Ga‐PTA treated aging group showed no significant elevation than the aging group at week 16 (Figure 3D). Thus, we speculated that the hindered nerve repair in aged rats was associated with cellular phenotype changes in Schwann cells.

Previous studies and our aforementioned results have indicated that Schwann cells undergo reversibly change to match the temporary exposure to a ferroptosis stimulation [27]. In senescent organisms, age‐related iron accumulation and antioxidant system deficiency exacerbates ferroptosis activity at the region of neurodamage, ultimately leading to the chronic long‐term exposure of ferroptosis‐related stressor [28]. When ferroptosis‐related stressor prolonged its duration, cells could be induced to undergo a long‐term or permanent change in cellular phenotype and a wide range of structural changes to molecules [21]. GO and KEGG pathway enrichment analysis demonstrated that Schwann cells in regenerative nerves from aged rats accentuate alterations in senescence‐associated secretory phenotype (SASP)‐related enrichment profiles (cellular senescence, cAMP signaling pathway, MAPK signaling pathway, and mTOR signaling pathway) compared with aging controls (Figure 3E). Consistently, SASP‐related genes cycling dependent kinase inhibitor 1a (Cdkn1a) and C‐C motif chemokine ligand 2 (Ccl2) exhibited marked upregulation in Schwann cells of aged rats with nerve injury (Extended Data Figure S3D). We next performed SA‐β‐gal staining on the RSC96 cell senescence models after Ga‐PTA intervention for 48 h. SA‐β‐gal is a lysosomal enzyme accumulated in the body with age. Compared with the senescent cell group, the rate of SA‐β‐gal positive staining cells and dyeing depth did not decrease remarkably in the Ga‐PTA intervened group, suggesting that using Ga‐PTA alone may not be able to modify the SASP expression in Schwann cells caused by the long‐term ferroptosis stimulation (Extended Data Figure S3C).

To elucidate the association between SASP expression and cellular phenotype changes in Schwann cells, we used ‘monocle3’ pseudotime analysis to simulate the dynamic developmental trajectory of Schwann cells in the aging group (Extended Data Figure S4A). We observed that dedifferentiated Schwann cells were located at the start of the trajectory, followed by a transition to non‐myelinating Schwann cells, with myelinating Schwann cells positioned at the end of the trajectory (Figure 3F, Extended Data Figure S4B). It is known that injury could induce the transition of myelinating and non‐myelinating Schwann cells into dedifferentiated Schwann cells in the sciatic nerve. During the nerve regeneration process in normal adult rats, dedifferentiated Schwann cells migrate across the injury site and redifferentiate into myelinating and non‐myelinating Schwann cells to facilitate repair. The failure of this subtype transition may result in inadequate Schwann cell filling in the injury gap, ultimately hindering the completion of the repair process [29]. Analysis of gene expression differences along the trajectory revealed the significant changes in the expression levels of SASP‐related genes during the transition from dedifferentiated Schwann cells to non‐myelinating and myelinating Schwann cells in the aged rats (Figure 3G, Extended Data Figure S4D). We further conducted CCL2, EGFR and JUN (key node genes in the SASP signaling network) projection on the trajectory and found that these were overexpressed in the Schwann cell redifferentiating process (Extended Data Figure S4C). Moreover, we measured the expression of CCL2 on the injured nerves by immunofluorescence. The fluorescence intensity of CCL2 was significantly increased in the injured nerves of aged rats but no significant reduction of the fluorescence intensity of EGFR was observed after Ga‐PTA nanoparticle intervention (Figure 3H). Gene enrichment analysis indicated that the levels of SASP‐related core genes (EGFR, LGFBP6 and JUN) and ferroptosis were both most prominently elevated during the C5 stage (the redifferentiation stage of Schwann cells) (Figure 3I). To track the contribution of SASP expression and ferroptosis level to the Schwann cell subtype adjustment, we interrogated published single‐cell embryonic limb profiles from PCW5 to PCW9 and annotated the sciatic nerve region based on S100B gene [30]. As studies of aging rat samples were few, we accessed data from human fetal limb samples as substitution. We used matched orthologues to align datasets of rats and humans from published studies, and found highly conserved cell composition and similar developmental trajectories between humans and rats (Extended Data Figure S5). The change of gene expression pattern of the developing limb demonstrated an enrichment of genes involved in SASP expression and ferroptosis pathway with age (Figure 3J, Extended Data Figure S4E, F). Meanwhile, the signature genes of mSC (such as MPZ and PRX) were downregulated in the sciatic nerve regions, whereas the signature gene of dSC (such as NGFR) was upregulated (Extended Data Figure S4G). These results suggest that, apart from serving as temporary stressor, excessive ferroptosis in the injury sites of aged sciatic nerves also acted as a chronic stressor to impede the Schwann cells redifferentiation to mSC after injury, ultimately impairing the repair process (Figure 3K). Thus, the more complex ferroptosis stimulation microenvironment in vivo could not be resolved solely by the application of Ga‐PTA nanoparticles.

### 2.4 Mmsc Treatment Restarts the Redifferentiation Program of Schwann Cells in the Injured Nerve of Aging Rats

**FIGURE 4:**
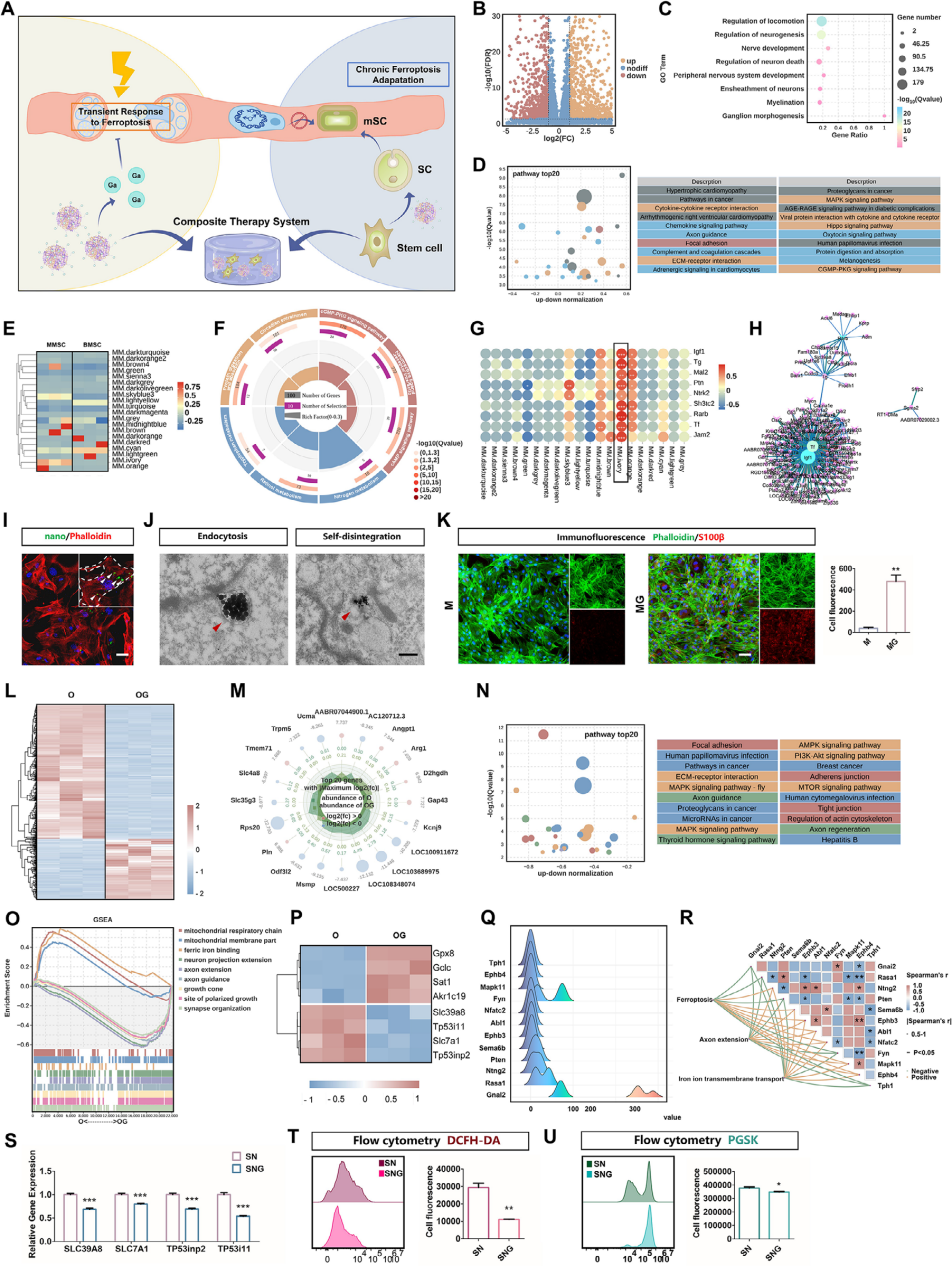
Mmsc treatment restarts the redifferentiation program of Schwann cells in the injured nerve of aging rats. (A) Schematic illustration of the effects of the synergistic system of Mmscs and Ga‐PTA nanoparticles. (B) Volcano plots of differentially expressed genes in Mmscs and Bmscs. (C) GO term analysis indicating a enrichment of pathways related to Schwann cell differentiation and neurodevelopment promotion potential in Mmscs. (D) KEGG pathway analysis of the top 20 enriched signaling pathways in Mmscs. (E) Module‐sample expression pattern for WGCNA co‐expression modules in Mmscs and Bmscs. (F) Enriched GO terms within the MM.ivory module. (G) The statistical significances of genes related to Schwann cell differentiation potential in each co‐expression module. (H) Integrated model of biomolecular interaction networks for Schwann cell differentiation potential‐related genes. (I) Nanoparticles were labeled with FITC (green) and incubated with Mmscs for 24 h. The immunofluorescence images of the endocytosis of Ga‐PTA nanoparticles in Mmscs. Scale bar = 25 µm. (J) The TEM images of the endocytosis and disintegration of Ga‐PTA nanoparticles in Mmscs. Scale bar = 500 nm. (K) The immunofluorescence images of S100B (green) and actin cytoskeleton (red) in Mmscs after Ga‐PTA intervention and its quantification data. Scale bar  =  50 µm. (L) Heatmap of gene expression profile in senescent PC12 cell models co‐cultured with GelMA‐Matrigel hydrogel loaded with Mmscs and Ga‐PTA nanoparticles. Cutoff line: Log2 fold change (Log2FC) > |2| and adjusted *p* value < 0.01. (M) Differential gene radar chart for in senescent PC12 cell models co‐cultured with the composite hydrogel versus Control. (N) KEGG pathway analysis of the top 20 enriched signaling pathways in senescent PC12 cell models co‐cultured with the composite hydrogel. (O) GSEA of GO analysis in senescent PC12 cell models co‐cultured with the composite hydrogel. (P) Heatmap of key genes contained in ferroptosis pathway in senescent PC12 cell models co‐cultured with the composite hydrogel. (Q) Ridgeline charts of differential genes contained in axon extension pathway. (R) Mantel test between the correlation matrix of the chosen highly expressed genes and key enrichment terms. (S) RT‐qPCR analysis for gene expression of ferroptosis‐related genes. (T) The intracellular ROS levels of senescent PC12 cell models co‐cultured with the composite hydrogel evaluated by flow cytometry. (U) The intracellular ferrous ion levels of senescent PC12 cell models co‐cultured with the composite hydrogel evaluated by flow cytometry. *p < 0.05, **p < 0.01, ***p < 0.001. M, Mmscs; MG, Ga‐PTA intervened Mmscs; SN, senescent PC12 cell models; SNG, Ga‐PTA‐intervention senescent PC12 cell models.

To overcome the failure of SASP‐secreting Schwann cell redifferentiation that hinders the nerve repair process, we isolated stem cells from the offspring of the aged recipient rats as seed cells and induced their differentiation into Schwann cells (Figure 4A). Through transcriptome sequencing (RNA‐seq) comparing Bmscs (bone marrow stem cells) and Mmscs, we identified distinct differentially expressed genes and pathways among the two cell types. Compared to Bmscs, Mmscs exhibited a significant enrichment of pathways related to Schwann cell differentiation potential and neurodevelopment promotion (Figure 4B–D). Weighted Gene Co‐expression Network Analysis (WGCNA) was conducted to distinguish functional modules among the differentially expressed genes between the two groups of cells, with clustering heatmap results indicating that the MM.ivory module was predominantly expressed in Mmscs (Figure 4E). Clustering analysis and gene enrichment analysis suggested that this module is highly associated with stem cell neural differentiation, and the gene interaction network within the module indicated that the regulatory core may involve the Igf1 and Tf genes (Figure 4F–H). These high‐throughput sequencing results suggest that Mmscs may possess superior Schwann cell differentiation potential. After co‐culture in vitro with Ga‐PTA nanoparticles for 24 h, Mmscs can form vesicles to endocytosis and subsequent disintegration these nanoparticles within the cells (Figure 4I,J). Considering that excessive concentration of Ga^3+^ might cause damage to seed cells, the working dose of Ga‐PTA was controlled based on the results of the calcein‐AM/PI staining (Extended Data Figure S6A,B). Immunofluorescence staining of the Schwann cell marker S100β evidenced that intervention with 25nM Ga‐PTA nanoparticles for 12 days could promote the differentiation of Mmscs into Schwann cell‐like cells, while the effect on promoting Schwann cell differentiation in Bmscs was not significant (Figure 4K, Extended Data Figure S6C). In addition, co‐culturing with Ga‐PTA nanoparticles could maintain the rate of SA‐β‐gal positive staining cells and staining depth in the Mmscs at a minimal level in senescence‐inducing conditions (Extended Data Figure S6D). This might imply a synergistic effect of Mmscs applied in the injured site in conjunction with Ga‐PTA nanoparticles.

We further explored the effects of the synergistic system of Mmscs and Ga‐PTA nanoparticles on the hydrogen peroxide‐induced senescent neural precursor cell models (Figure 4A). The senescent models of PC12 cell lines were co‐cultured with gelatin methacryloyl (GelMA) ‐Matrigel hydrogel loaded with Mmscs and Ga‐PTA nanoparticles for RNA‐seq. Clustering heatmap results indicated that massive differentially expressed genes emerged in the senescent PC12 cell models following intervention with this composite system (Figure 4L). Genes associated with neural regeneration were highly enriched post intervention, while the expression levels of ferroptosis‐related genes were decreased (Figure 4M). The top 20 significantly enriched KEGG pathways indicated that axon guidance, axon regeneration, mitogen‐activated protein kinase (MAPK) signaling and several cell migration‐related pathways exhibited differential expression in the Ga‐PTA intervention group (Figure 4N). And the GSEA analysis of GO database verified that neural regeneration‐related pathways were remarkably enriched in the Ga‐PTA intervention cells, while ferroptosis‐related pathways were depleted (Figure 4O). Expression of key genes involved in these pathways were in consistent with the above results (Figure 4P,Q). Furthermore, in the mantel test between the correlation matrix of the chosen highly expressed genes and the top enrichment terms, we found that ferroptosis signaling pathway was negatively correlated with neural regeneration‐related genes (Figure 4R). Subsequently, RT‐qPCR analysis validated that the expression levels of key ferroptosis‐related genes were significantly changes following treatment with this composite system (Figure 4S). And flow cytometry analysis demonstrated that the intervention effectively reduced ROS levels and ferrous ion intensity within the senescent neural precursor cell models (Figure 4T,U). We further compared the cell viability between the senescent PC12 models before and after intervention, and found that the cell viability was increased after co‐culturing with the composite system (Extended Data Figure S7A). Meanwhile, the wound healing experiments showed that the addition of the system promoted the migration of senescent PC12 cells (Extended Data Figure S7B).

### 2.5 Construction of a Composite Nerve Conduit Carrying Ga‐PTA Nanoparticles and Mmscs

**FIGURE 5:**
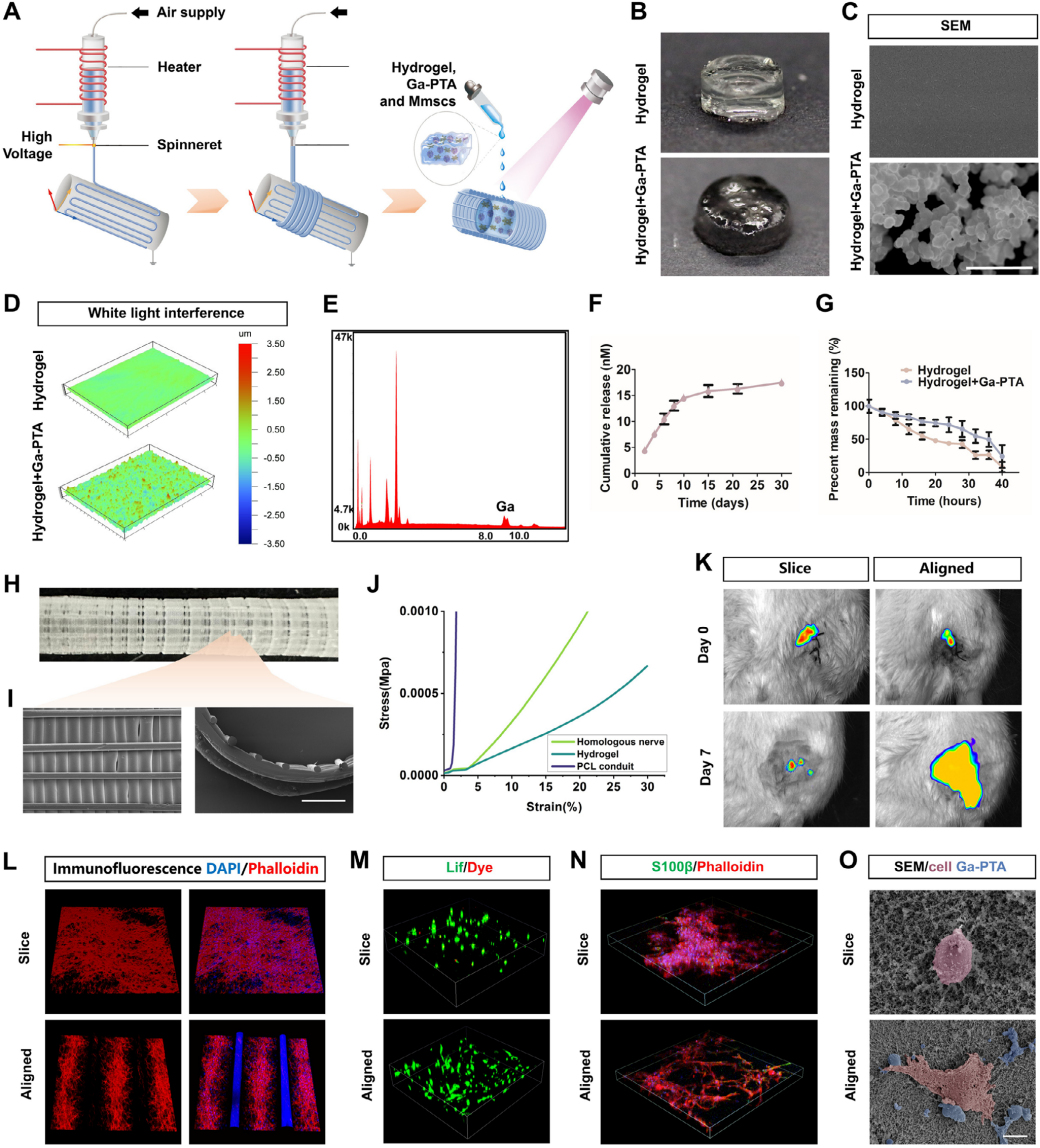
Construction of a composite nerve conduit carrying Ga‐PTA nanoparticles and Mmscs. (A) Preparation of the composite nerve scaffold. (B) Representative photographs of Matrigel modified GelMA hydrogel and Matrigel‐GelMA hydrogel incorporated with Ga‐PTA nanoparticles. (C) Representative SEM images of Matrigel‐GelMA hydrogel incorporated with Ga‐PTA nanoparticles. Scale bar = 300 nm. (D) The white light interference images of Matrigel‐GelMA hydrogel incorporated with Ga‐PTA nanoparticles. (E) EDS spectrum of Matrigel‐GelMA hydrogel incorporated with Ga‐PTA nanoparticles. The peak signals of gallium is marked. (F) The sustained release curve of Ga^3+^ of the hydrogel scaffolds. (G) The degradation performance of the hydrogel scaffolds. (H) Fabrication of the nerve conduits with precisely aligned intraluminal microfibers and external supporting fibers by melt electrowriting (MEW). (I) Representative SEM images of the nerve conduits. Scale bar = 400 µm. (J) Young's modulus of natural nerves, the composite hydrogel and the nerve conduit. (K) The permeability of the nerve conduits in the internal environment. (L) Immunofluorescence images of the cell skeleton (red) of Mmscs on the surface of the scaffold. (M) Three‐dimensional superposition of the LCSM images of cell viability of Mmscs within the nerve conduit. (N) Three‐dimensional superposition of the LCSM images of the cell skeleton (red) and S100β (green) of Mmscs within the nerve conduit. (O) Representative SEM images of Mmscs within the nerve conduit. Scale bar = 3 µm. *p < 0.05, **p < 0.01, ***p < 0.001. Slice, scaffolds made by PCL slice; Aligned, aligned fiber scaffolds fabricated by melt electrospinning writing.

After uniformly mixing Ga‐PTA nanoparticles with the Matrigel modified GelMA hydrogel (ratio of Matrigel: GelMA is 5:1, the optimal proportion determined by preliminary experiments), ultraviolet light was used for curing (Figure 5A, Extended Data Figure S8A,B). The black color indicated the successfully incorporated of Ga‐PTA nanoparticles in the hydrogel (Figure 5B). Thereafter, the morphology of the cross‐sectional view of the composite hydrogel was observed by SEM. It could be observed that Ga‐PTA nanoparticles were abutting uniform spheres inside the composite hydrogel (Figure 5C). White light interference of the composite hydrogel containing Ga‐PTA nanoparticles indicated that the roughness of the hydrogel was greater than that of the GelMA‐Matrigel composite hydrogel (Figure 5D). And energy dispersive spectroscopy (EDS) results showed the presence of gallium element peaks in the composite hydrogel (Figure 5E). At 37°C, this composite hydrogel can slowly release Ga^3+^ in a neutral liquid environment and completely degradate within 2 days to avoid obstructing regenerating nerve fibers (Figure 5F,G).

To further evaluate the repair of peripheral nerve defects in aged rats after the composite system implantation, nerve conduits with precisely aligned intraluminal microfibers (diameter < 100 µm) and external supporting fibers (diameter > 100 µm, perpendicularly and densely arranged relative to the inner microfiber layer) was fabricated using melt electrowriting (MEW) for structural support (Figure 5H,I). This nerve conduit exhibited favorable mechanical properties, with a Young's modulus higher than that of natural nerves, capable of withstanding external pressure and preventing secondary damage from compressing regenerating nerves (Figure 5J). The permeability of the nerve conduits in the internal environment was estimated using Cy7‐labeled Ga‐PTA nanoparticles. 7 days post implantation, we observed more conducive transmural material exchange across the nerve conduits fabricated by MEW (Figure 5K). After mixing Mmscs with this composite hydrogel and curing it within the scaffolds, we obtained nerve conduits loaded with Mmscs and Ga‐PTA nanoparticles (Figure 5A). The radial and axial stiffness, maximum stress and maximum load of the conduits filled with the composite hydrogel was similar to that of empty conduits (Extended Data Figure S8C–F). Mmscs were cultured on the surface of the scaffold and the results showed that the aligned fibers on the conduit surface promoted the directional growth of Mmscs within the conduit (Figure 5L). After cell viability staining, confocal microscopy was employed to synthesize three‐dimensional images at different z‐axis positions, revealing that Mmscs exhibited good viability at various locations within the hydrogel (Figure 5M). Immunofluorescence staining of the cytoskeleton and S100β demonstrated that Mmsc cells displayed a more pronounced differentiation toward Schwann cell‐lineage in the nerve conduits with aligned fibers (Figure 5N). SEM analysis indicated that Mmsc cells showed better cellular extensibility within the aligned nerve conduits (Figure 5O).

### 2.6 Neurological Function Recovery, Nerve Regeneration and Remyelination of the Aged Rats in the Composite Nerve Conduits

**FIGURE 6:**
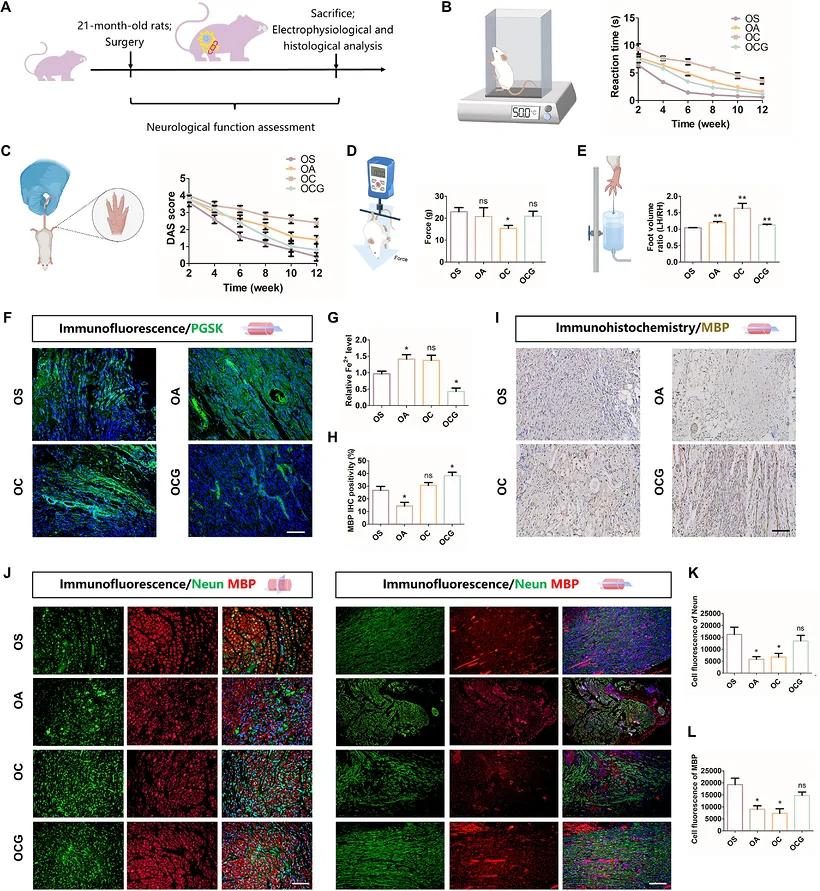
Neurological function recovery, nerve regeneration and remyelination of the aged rats in the composite nerve conduits. (A) Timeline of sciatic nerve defect construction and nerve conduit implantation. (B) The hindlimb withdrawal latency in response to thermal/cold stimuli. (C) Dynamic ankle score (DAS) assessment of the hindlimb. (D) Grip strength tests of the hindlimb at week 16 after the operation. (E) Paw edema tests of the hindlimb at week 16 after the operation. (F,G) Immunofluorescence staining for ferrous ion in the regenerated nerves from the aged rats intervened with the composite nerve conduits. Quantitative analysis of intensity is also shown. Scale bar = 50 µm. (H,I) The images of immunohistochemistry staining of MBP. n = 3 per group. Quantitative analysis of intensity is also shown. Scale bar = 50 µm. (J–L) Immunofluorescence images for the localization and the expression of Neun (green), MBP (red) and DAPI (blue). n = 3 per group. Quantitative analysis of intensity is also shown. Scale bar = 50µm. *p < 0.05, **p < 0.01, ***p < 0.001. OS, aging sham group; OA, aging autologous nerve anastomosis group; OC, aging PNI group treated with Mmsc‐loaded nerve conduits; OCG, aging PNI group treated with Ga‐PTA and Mmsc‐loaded nerve conduits.

To evaluate the repair efficacy of these conduits on injured nerves in aged rats, we established 15‐mm sciatic nerve defect models in 21‐month‐old Sprague‐Dawley (SD) rats and implanted the composite conduits (Figure 6A). The sham group, autologous nerve graft group and neural conduit with Mmscs group were set as controls in aged rats. Postoperative observation of hindlimb withdrawal latency in response to thermal/cold stimuli demonstrated that the composite conduit group achieved sensory functional recovery comparable to the aging sham group at 12 weeks (Figure 6B). Dynamic ankle score (DAS) assessment of neuromuscular reinnervation showed that the composite conduit group reached similar scores to the sham group at 10 weeks post‐injury (Figure 6C). Consistently, grip strength tests at 16 weeks revealed comparable paw grip force between the composite conduit and sham groups (Figure 6D). Paw edema tests at 16 weeks also indicated that composite conduit treatment effectively alleviated injury‐induced hindlimb swelling (Figure 6E).

At 16 weeks post‐operation, sciatic nerve samples were collected and subjected to HE staining, TB staining, immunohistochemistry (IHC), and immunofluorescence (IF) to evaluate nerve regeneration of aged rats. HE staining results showed that the regenerated nerve fibers arranged tightly and regularly with favorable healing and less inflammatory cell infiltration in the composite conduit group (Extended Data Figure S9A). TB staining showed that the composite nerve conduit group contained similar cross‐sectional diameters of the regenerated nerve than the sham group. And the number of myelinated axons and density of Nissl bodies of the regenerated nerve fiber bundles in the composite nerve conduits were comparable to the sham group, which were superior to the other groups (Extended Data Figure S9B). PGSK fluorescent probe staining revealed significantly reduced ferroptosis levels in the injured nerves of aging rats treated with composite conduits (Figure 6F,G). IHC results indicated the densely distributed myelin sheath and markedly increased MBP‐positive area ratios in cross‐sections of regenerated nerves from the composite conduit group (Figure 6H,I). Consistently, IF analysis of transverse sections showed similar NeuN (labeled neuronal cells)‐ and MBP‐positive area ratios between the composite conduit and sham groups. And the images of longitudinal section showed that the myelinated nerve fibers were parallel‐aligned in the composite conduit and sham groups, along with larger positive expressed areas of MBP and NeuN than the other groups, suggesting that the composite therapy might help restoring the aging affected decline of nerve tissue repair (Figure 6J–L).

### 2.7 Gastrocnemius Muscle Reinnervation Post Implantation of the Composite Nerve Conduits

**FIGURE 7:**
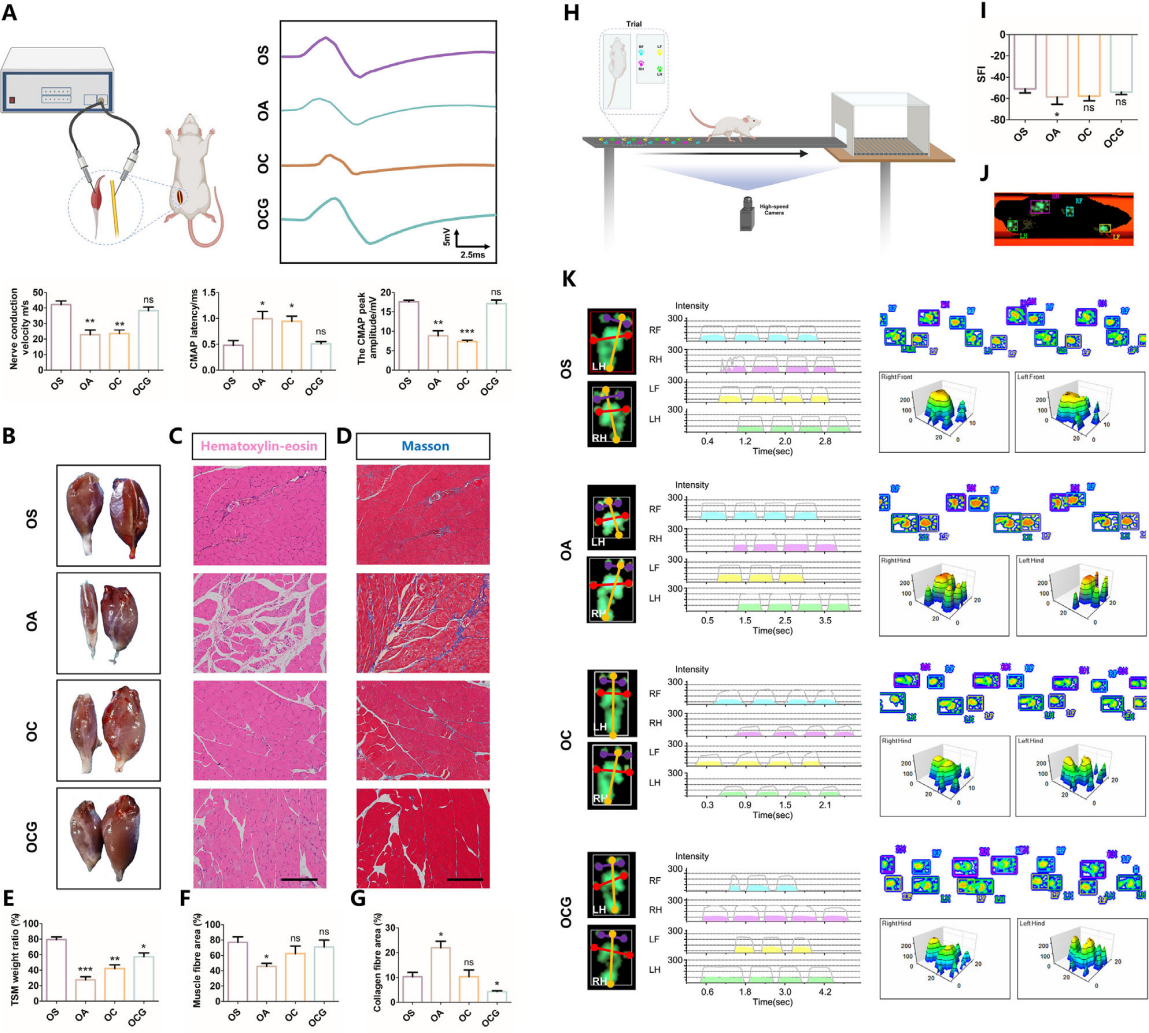
Gastrocnemius muscle reinnervation post implantation of the composite nerve conduits. (A) Electrophysiological assessment of CMAP for different aspects (the NCV, the latency period of CMAPs, and the peak amplitude of CMAPs). (B, E) The gross anatomical images of the gastrocnemius muscles of bilateral hindlimbs. Left is the gastrocnemius on the experimental side. Statistical analysis of wet weight recovery is also shown. (C, D, F, G) HE and Masson's trichrome staining images of cross‐sectional gastrocnemius muscle on the operated side. The quantitative analysis of muscle fibers area and collagen fibers area is also shown. Scale bar = 200µm. (H–K) Functional recovery analysis via catwalk gait. The SFI is also evaluated. *p < 0.05, **p < 0.01, ***p < 0.001. OS, aging sham group; OA, aging autologous nerve anastomosis group; OC, aging PNI group treated with Mmsc‐loaded nerve conduits; OCG, aging PNI group treated with Ga‐PTA and Mmsc‐loaded nerve conduits.

Electrophysiological evaluation of regenerated nerves post‐treatment demonstrated that the composite conduit group exhibited electrical signal conduction parameters comparable to the aging sham group, with NCV at 38.3 ± 4.164 m/s, CMAP amplitude at 17.25 ± 1.652 mV, and CMAP latency at 0.56 ± 0.074 ms, versus autograft values of 41.81 ± 4.693 m/s NCV, 17.76 ± 0.598 mV CMAP amplitude, and 0.49 ± 0.168 ms CMAP latency. These metrics were significantly superior to the other two groups, indicating restored neural conductivity in the composite conduit group (Figure 7A).

The gastrocnemius muscle was a distal target of sciatic innervation. Gross views and wet weight ratios of the gastrocnemius muscle showed a less severely gastrocnemius atrophic and a much higher ratio of the surgical‐to‐contralateral wet weight recovery in the composite conduit and the sham group compared to other controls (Figure 7B,E). HE and Masson's trichrome staining further revealed more ordered muscle fiber alignment and reduced collagen deposition in the composite conduit‐treated group (Figure 7C,D,F,G). Further, immunofluorescence staining of myosin heavy chain (MYH) showed the composite conduit‐treated group achieved the most robust protective effect on skeletal muscle (Extended Data Figure S9C).

Further CatWalk gait analysis demonstrated restored footprint area and spatiotemporal pressure distribution in the surgical hindlimb (left) of aged rats treated with composite conduits, comparable to the contralateral side (Figure 7H,J,K). And the composite conduit group showed similar sciatic functional index (SFI) values to the sham group, indicating the positive role of the composite therapeutic system to improve motor capacity and function recovery in aging PNI rats (Figure 7I).

Considering the histotoxicity of Ga^3+^, we evaluated the biological safety of the composite nerve conduit in vivo. The results of blood routine analysis results of rats in the composite nerve conduit group and the sham group generally displayed little difference, suggesting that the implantation will not cause severe inflammation and blood disease after implantation (Extended Data Table S1). Additionally, histopathology analysis of the heart, liver, spleen, lung, and kidney confirmed that the composite nerve conduits did not induce remote organ toxicity and had good biocompatibility during the 16‐week in vivo application (Extended Data Figure S10).

In summary, excessive ferroptosis at the nerve injury site in aged rats leads to transient declines of Schwann cell function, along with chronic acquisition of the SASP and difficulty in re‐differentiation post Injury‐triggered dedifferentiation in Schwann cells. This study developed a composite nerve conduit with Ga‐PTA nanoparticles and Mmscs dual effect: the Ga‐PTA nanoparticles limited ferroptosis and provided a favorable regenerative microenvironment, whereas the Mmscs restarts the redifferentiation program of Schwann cells for nerve tissue repair.

## 3 Discussion

Our findings establish that the impaired peripheral nerve regeneration in aged rats stems from dual ferroptosis‐mediated pathologies: transient exposure to ferroptotic stressors compromises Schwann cell proliferation and migration, while chronic exposure disrupts reparative mechanisms in senescent Schwann cells. Upon investigating these potential mechanisms, we innovatively engineered a composite nerve guidance conduit loaded with Ga‐PTA nanoparticles and Mmscs, and demonstrated the efficiency of the nerve conduit on the peripheral nerve defect repairing in aged rats.

Tissue repair and regeneration are critical biological processes that resile organ functionality post‐injury, yet these capacities become progressively attenuated with advancing age [31, 32]. Previous studies have recognized the age‐related declines in nerve regenerative capacity. The progressive slowing of nerve regenerative capacity of the aging mammals is not limited by neuronal intrinsic growth responses, but is profoundly influenced by the altered SC responses [7, 16, 33]. Our results demonstrated the aging‐related insufficient cell function and alterations in the subtype composition of Schwann cells ultimately manifesting as impaired myelinated fiber regeneration and suboptimal functional recovery in aged rats. Under nerve defect stimuli, Schwann cells dedifferentiate into a phenotype exhibiting stem‐like plasticity and then undergo a phenotypic reprogramming process that resembles redifferentiation to initiate the nerve repair state [34, 35]. Given the major transcriptional and phenotypic changes that occur in Schwann cells after injury, it is predictable that these cells may be more vulnerable to age‐acquired alterations. In the lesion site of aging rats, we have demonstrated that these cells occupied limited proliferative and migrative capabilities, slowing down the Büngner band elongation. Furthermore, these dedifferentiated Schwann cells also exhibited an impaired ability to redifferentiate into mSCs, which could ensheathe regenerating axons and eventually lead to the formation of mature myelinated nerve fibers. Still, it remains to be determined whether the altered SC responses result entirely from extrinsic factors derived from the neural regenerative microenvironment of aging rats or age‐acquired intrinsic defects.

Here we established the dysregulated ferroptosis pathway as an underlying driver of this aging‐dependent nerve regenerative impairment and defined influence of ferroptosis pathway to Schwann cells during this process. Ferroptosis, an iron‐dependent programmed cell death modality, is characterized by iron overload and ROS accumulation [36]. Previous studies have demonstrated that PNI sites exhibit elevated ferroptosis levels compared to non‐injured regions, attributable to iron release from damaged microvasculature and erythrocyte degradation [37]. In aging organisms, glutathione depletion exacerbates intracellular iron accumulation at injury sites, aggravating in pathological iron dyshomeostasis [38]. Our data has demonstrated that the microenvironment with high ferroptosis levels exerts an inhibitory effect on the proliferation and migration capabilities of Schwann cells in the regenerating nerves. And the function of the senescent Schwann cell models could be improved by reducing the level of ferroptosis signaling in the culture environment. Interestingly, we found that Schwann cells in the injured site of regenerating nerves exhibited partly distinct cellular fate impairment compared to that of cell culture models and the impairment could not be alleviated after ferroptosis inhibition. Hence, this divergence may not be fully explained by the inhibitory effect of the regenerative microenvironment and may arise from the alterations in inherent differences in the cellular characteristics of Schwann cells.

In senescent cells, the iron homeostatic mechanisms alter to afford protection from the excessive iron accumulation‐mediated oxidative stress [39]. Previous study have confirmed that iron accumulation in senescent cells was associated with autophagic/lysosomal dysfunction with no possibility of splitting the accumulate iron by cell division. The abnormally high levels of labile ferrous iron in lysosomes could drive the senescence‐associated ROS and fuels the generation of SASP [40]. Moreover, it was demonstrated that treatment of senescent cells with an iron chelator efficiently suppresses the SASP, thereby mechanistically connecting damage‐initiated iron uptake with the SASP. The dysfunction of the cell proliferation and differentiation capacity of senescent cells is thought to be primarily driven by their secretion of the unique mix of proinflammatory factors termed the SASP [41]. Our results also established that the persistent intracellular ferroptosis stressor accumulation could accentuate the SASP expression in Schwann cells in aged rats with peripheral nerve trauma. This pathological state disrupts the redifferentiation trajectory and repair programs of Schwann cells within the critical regenerative time window, ultimately compromising the establishment of ordered myelinating phenotypes. And the repair program of the senescent Schwann cells failed to be fully restored after ferroptosis inhibition. Transplantation of exogenous Schwann cells that have not undergone senescence‐associated intrinsic alterations represents a potential therapeutic alternative. Nevertheless, previous studies have identified critical limitations: donor cell scarcity, technical challenges in isolation/expansion, rapid functional decline post‐implantation (reduced proliferation/secretory capacity), and significant host immune rejection [42]. Directed differentiation of stem cells into Schwann lineages offers a promising strategy to replenish functional glial populations for neurotrophic factor secretion, myelination acceleration, and axonal elongation [43]. Through high‐throughput sequencing and in vitro differentiation assays, we discovered that Mmscs isolated from the offspring of the aged recipient rats exhibited superior Schwann‐lineage differentiation potential and lower host immune rejection. Crucially, the transplantation of Mmscs to the nerve defect significantly improved myelination and promoted nerve regeneration of aging rats. Thus, we proposed that localized ferroptosis overload not only induced transient suppression of proliferative and migratory capacities in neural cells at injury sites, but also triggered chronic oxidative stress exposure in Schwann cells, culminating in irreversible damaging adaptations.

While a growing number of research has focused on age‐related declines in regenerative potential, current investigations predominantly conceptualize it as a unidirectional and irreversible process, resulting in a paucity of mechanism‐based tissue engineering strategies to enhance nerve regeneration in aging organisms [44]. We engineered a ferroptotic stimuli‐responsive Ga^3+^‐release nanoparticles via a modified Stöber method [45]. The responsiveness is rooted in the chemical design of the nanoparticles as a metal‑phenolic coordination network formed between the galloyl groups of poly (tannic acid) (PTA) and Ga^3^^+^ ions. Under normal physiological conditions with basal ferroptotic stress, this coordination structure remains stable, and the nanoparticles maintain their integrity with minimal Ga^3^^+^ leakage. However, when the microenvironment is enriched with ferrous ions (Fe^2^^+^) and reactive oxygen species, hydroxyl radicals (·OH) generate via the Fenton reaction (Fe^2^^+^ + H_2_O_2_ → Fe^3^^+^ + OH^−^ + ·OH). These highly reactive ·OH radicals attack the phenolic hydroxyl groups of the galloyl moieties, oxidizing them to phenoxyl radicals. This oxidative modification weakens the galloyl‐Ga^3^^+^ coordination bonds, leading to disintegration of the nanoparticle network and consequent release of Ga^3^^+^ in a ferroptotic‑stimuli‑triggered manner. Thus, the responsiveness is governed by the synergistic presence of Fe^2^^+^ and ROS, rather than pH or other single factors, ensuring that the nanoparticles remain inert under normal conditions while achieving targeted Ga^3^^+^ release specifically within the ferroptosis‑rich lesion microenvironment. Functional validation of these nanoparticles in ROS‐induced senescent Schwann cell models demonstrated the nanoparticles significantly attenuated ferroptosis markers while enhancing cellular proliferation and migration capacities. Notably, Mmsc underwent efficient Schwann cell differentiation when co‐cultured with our ferroptosis‐responsive self‐disintegrating nanoparticles. Ferroptosis‐responsive self‐disintegrating nanoparticles and Mmscs were encapsulated within the GelMA‐Matrigel hydrogel to form a composite therapeutic system. And the composite therapeutic system demonstrated significant ferroptosis mitigation and microenvironmental remodeling capabilities when co‐cultured with the senescent neuronal precursor cell PC12 models.

To address mechanical instability of hydrogel carriers under physiological stress during rodent mobility, we engineered a novel nerve conduit to load the composite hydrogel. It has been confirmed that Schwann cell migration and axonal regrowth from both stumps requires stringent topographical guidance, as misdirected extension due to aberrant environmental cues severely compromises functional recovery [46]. The directional guidance requires that the aligned fibers within the nerve conduit have a diameter of less than 100 µm (approaching cellular dimensions), demanding high precision in material structure [47]. Thereby, we fabricated the nerve conduits with precisely aligned intraluminal microfibers (diameter < 100 µm) and external supporting fibers (diameter > 100 µm, perpendicularly and densely arranged relative to the inner microfiber layer) using MEW technology. The nerve conduit emulated the structural features of the natural epineurium, whose aligned microfibers closed to the size of cells providing anisotropic guidance for Schwann cells in the nerve defect. Concurrently, the nerve conduit could prevent infiltration of surrounding tissue into the neural regeneration site while maintaining appropriate conduit permeability to avoid the exacerbation of ferroptosis caused by the excessive accumulation of metabolic products within the conduit. Under the induction of high ferroptosis levels at the nerve injury site, intraluminal Ga‐PTA nanoparticles disintegrated and competitively inhibited excessive ferroptosis to establish a pro‐regenerative microenvironment. Meanwhile, Mmscs was induced to progressively differentiate into Schwann cell‐like cells which overcame the impaired redifferentiation capacity caused by resident Schwann cells with SASP after a relatively long period of time. Ultimately, the aligned guidance fibers within the conduit directed the neoformed Schwann cell cords across the lesion gap, thereby repairing the peripheral nerve defects in aging rats.

In conclusion, our study establishes the dysregulated ferroptosis pathway as an underlying driver of this aging‐dependent nerve regenerative impairment and defines the dual influence of ferroptosis pathway to Schwann cells during this process. This discovery provides theoretical insights for promoting nerve regeneration of aging individuals. Nevertheless, there are still several limitations of this study. First, the further identification of key targets through which the implanted cells exert their therapeutic effects is required to enable future interventions at these targets to circumvent the need for cell implantation. Second, autologous Mmscs might represent a better option despite the application was still limited by the available extraction and preservation techniques in rats at present. It may be possible in the future to store Mmscs from rats in their youth in Biobank for later application in their old age. Ultimately, an extended types of senescent animal models and nerve injury paradigms is needed to validate the generalizability of the proposed mechanisms and regenerative strategies.

## 4 Materials and Methods

### 4.1 Animals and Materials

All rat protocols and experiments were approved by the Zhejiang University Medical School Animal Care and Use Committee, and the Zhejiang Academy of Medical Sciences Animal Care and Use Committee (approval number X1901457). Sprague‐Dawley rats (6–8 weeks old, male) were purchased from the Experimental Animal Center of the Zhejiang Academy of Medical Science. The aged Sprague‐Dawley rat models (21 months old, male) were purchased from the Shanghai Laboratory Animal Center. All animals were grouped and housed with a 12 h light/dark cycle and 50%–70% humidity at room temperature, with free access to water and standard autoclaved chow.

Tannic acid, gallium (III) nitrate hydrate and PCL (average Mn = 10 000) were purchased from Sigma‐Aldrich (USA). Formaldehyde was obtained from TEA, Sinopharm Chemical Reagent Co., Ltd. (China) and ammonia solution was purchased from Aladdin (China). The MEW printer (EFL‐MDW5800, Suzhou Intelligent Manufacturing Research Institute, China) was used to fabricate the microtopographies of PCL constructs, and the specific printing steps were based on a previous research [48]. The cell line RSC96 (GF1900C) and PC12 (CRL‐1721) were purchased from the American Type Culture Collection (ATCC, USA). Dulbecco's modified Eagle's medium (DMEM), fetal bovine serum (FBS), horse serum and penicillin/streptomycin (100 U/mL penicillin and 100 µg/mL streptomycin, P/S) were derived by Gibco (USA). GelMA was provided by the EFL Suzhou Intelligent Manufacturing Research Institute and (China) and Matrigel basement membrane matrix was purchased from Corning (USA). The 30% hydrogen peroxide stock solution was obtained from Sigma‐Aldrich (USA). The live/dead staining kit was derived from Invitrogen (USA). β‐galactosidase assay kit was purchased from Solarbio (China) and CCK8 reagent was derived from GLPBIO (USA). Superscript II first‐strand cDNA synthesis kit was purchased from TaKaRa (Japan). Rat‐Piezo1‐shRNA, GMPO polybrene, and puromycin dihydrochloride were derived by Shanghai GenePharma Co. (China). Superscript II first‐strand cDNA synthesis kit was purchased from TaKaRa (Japan). Rat‐Piezo1‐shRNA, GMPO polybrene and puromycin dihydrochloride were derived by Shanghai GenePharma Co. (China). And all the primary antibodies except MHC (Santa Cruz Biotechnology, USA) were purchased from Abcam (China). The fluorescent FITC dye, fluorescent Cy7 dye and fluorescent probe Phen Green SK (PGSK) were derived from GlpBio (USA). DCFH‐DA probe was purchased from Sigma‐Aldrich (USA).

### 4.2 Synthesis of Ga‐PTA Nanoparticles

NH3̇H2O (0.1 mL, 25 wt%) was added into a 20mL solution containing absolute ethanol and water (1:1 volume ratio). Subsequently, tannic acid (TA) (0.1 g) was added and continually stirred until completely resolution. By adding 0.2 mL of formaldehyde solution and 20 µL of biocompatible surfactant Pluronic F127 to the system and stirring at 30 °C for 24 h, TA molecules could assemble orderly to form PTA oligomers. The above‐obtained oligomers were immersed in Ga (NO3)3 solution (50% feed ratio) and stirred for 6 h to obtain Ga‐PTA nanoparticles. Next, the solution was transferred into an autoclave and heated at 150 °C for 24 h under a static condition to stabilize the as‐prepared nanospheres. After the temperature was cooled down, the samples were collected by centrifugation and dried at 60 °C.

### 4.3 Preparation of GelMA‐Matrigel Composite Hydrogel

The photo‐initiator LAP was added to PBS solution and incubated in a light‐protected water bath at 45°C for 15 min. The freeze‐dried GelMA foams were dissolved in the LAP solution and again heated in a 55°C water bath for 30 min under light‐protected conditions. Prechilled (at 4°C) Matrigel matrix was rapidly incorporated into the GelMA/LAP hydrogel solution and thoroughly mixed prior to cooling‐induced gelation. After irradiation for 60 s by ultraviolet light, the GelMA/Matrigel/LAP solution was crosslinked into GelMA/Matrigel/LAP hydrogel.

### 4.4 Fabrication of Nerve Scaffold With Aligned Microstructure

Nerve conduits with aligned microstructure were prepared using MEW technology. First, PCL was loaded into a syringe and heated above 75°C to form a homogeneous polymer melt. Then, melt electrowriting was conducted under precisely controlled height‐dependent deposition paths at a pushing pressure of 0.15 bar and a working voltage pf 4 kV. Specifically, a dual‐layer fibrous architecture with distinct diameters was fabricated: The outer layer consisted of supporting fibers (diameter = 200 µm) arranged circumferentially in a dense, gapless configuration, while the inner layer oriented microfibers (diameter = 70 µm) aligned parallel to the longitudinal axis with 300 µm inter‐fiber spacing. The intersecting angle between the inner and outer layers was maintained at 90°.

### 4.5 Characterization

The X‐ray diffractometer (Bruker D8 Advance) was used to detect the structure of Ga‐PTA nanoparticles. The particle size and zeta potential of Ga‐PTA nanoparticles were tested using a nano particle potential analyzer (Zetasizer Nano ZS90), with a testing temperature of 25°C. The transmission electron microscope (TEM) and energy‐dispersive X‐ray spectrometer (HITACHI, Japan) were used to test the elemental composition and structure of Ga‐PTA nanoparticles. The inductively coupled plasma mass spectrometry (icp‐ms) characterized the endocytosis and absorption of Ga‐PTA nanoparticles of Schwann cells.

The cross‐sectional roughness of nanoparticle‐laden hydrogel was tested by white light interference scanning electron microscopy (Veeco, USA). The Ga^3+^ sustained‐release curve of hydrogels in PBS was measured by visible UV spectroscopy (AKOYA Vectra Polaris) at room temperature. The degradation rate of hydrogel in vitro was also tested. The freeze‐drying hydrogel was put into a 5 mL PBS solution and placed it in the shaker at 37°C. And every 4 h, the weight loss of the hydrogel was recorded.

Scanning electron microscope (SEM) was applied to observe the surface micromorphology of nerve scaffolds. Tensile strength of nerve scaffolds was measured using a universal testing machine (UTM‐2203). After the samples were broken, a stress–strain curve was recorded, and the radial stiffness, axial stiffness, maximum load and maximum stress were calculated.

### 4.6 Single Cell RNA Sequencing

CCI (or Sham‐CCI) surgery was performed on aged Sprague Dawley rats based on the method reported by predecessors [22]. 3 days after surgery, about 10 mm of the CCI ligated sciatic nerve was dissected from rats transcardially perfused with PBS and was immediately enzymatic dissociated. The purified cells were harvested and used to prepare scRNA‐seq libraries following manufacturer's instructions (10X Genomics). And the libraries were sequenced on an Illumina Novaseq 6000 platform. For previously published control Sprague Dawley rat (GSE216665) datasets, files downloaded from GEO (https://www.ncbi.nlm.nih.gov/) database were extracted and imported into a Seurat‐compatible object.

RNA‐seq data and sample annotation information were obtained using the Seurat R package (version 4.3.1). Cells with < 500 genes, > 5500 genes, or > 10% mitochondrial genes were filtered out. After normalizing scRNA‐seq data and controlling for the relationship between average expression and dispersion, highly variable genes were identified in each cell. Differentially expressed genes (DEGs) for a given cell type were determined with the FindAllMarkers function, and a UMAP was used to dimension reduction and classify the cells into different clusters.

### 4.7 Cell Culture and H_2_O_2_‐Induced Senescence

Rat neuronal Schwann cell line (RSC96) was cultured in high glucose Dulbecco's modified Eagle's medium/Nutrient Mixture F‐12 (DMEM/F‐12) supplemented with 10% fetal bovine serum (FBS) and 1% penicillin−streptomycin formulation at 37°C with 5% CO_2_. PC12 cells were cultured in high glucose DMEM with 10% horse serum, 5% FBS and 1% penicillin−streptomycin formulation. In order to induce senescent cell models, proliferating (p.4 or p.5) cells were treated with 50 µM exogenous H_2_O_2_ for 12 h and then kept on culture in fresh medium for 48 h.

### 4.8 Cell Viability Assays

After seeding the treated cells in 96‐well plates for 1, 2, 3, or 4 days, a Cell Counting Kit‐8 (CCK‐8, Beyotime, China) was applied to evaluate cell proliferative ability according to the manufacturer's instructions. Subsequently, the medium was collected from each well for further absorbance measurement at 450 nm and normalized to the reference. In addition, the cytotoxicity of different treatments was also measured using a Calcein‐AM/PI Viability/Cytotoxicity Kit (Solarbio, Beijing) according to the protocol provided by the manufacturer.

### 4.9 Senescence‐Associated β‐Galactosidase Staining

Cells were stained for Senescence‐associated β‐galactosidase (SA‐β‐gal) according to the manufacturer's protocol. After fixed for 15min at room temperature, cells were incubated overnight with SA‐β‐gal detection solution at 37°C without CO_2_. The blue‐stained senescent cells were captured under a Leica microscope.

### 4.10 Wound Healing Assay

Cells were suspended in cell culture medium at a density of 2 × 104 cells/ml and transferred to a mold chamber (ibidi, Germany) with a 1 mm wide insert. Insert was removed after cell reaching > 95% confluent to allow cell migration. Cleaned areas were captured using a microscope from Day 0 to Day 4 and measured using ImageJ software.

### 4.11 Ribonucleic Acid Isolation and Quantitative Reverse‐Transcription Polymerase Chain Reaction

Total RNA samples were isolated from treated cells using TRIzol reagent (Ambion, Inc., Austin, Texas, US) following manufacturer's protocol. We reverse‐transcribed 1 µg template RNA using a PrimeScript RT reagent kit (TaKaRa Bio, Shiga, Japan) and measured expression of the target gene using a SYBR Fast Universal qPCR Kit (Kapa Biosystems, Inc. [Roche Life Science, Basel, Switzerland]) for real‐time qPCR. Primer sequences are provided in Extended Data Table S2. We normalized the final calculated results to GAPDH and converted them using relative quantification (2‐ΔΔCt).

### 4.12 Flow Cytometry

Intracellular ROS content was measured by the flow cytometry utilizing DCFH‐DA. Cells were stained with 10 µM DCFH‐DA at 37°C for 30 min, then harvested and analyzed by the flow cytometry. PGSK probe was applied to detect the concentration of intracellular ferric irons according to the manufacturer's instruction.

### 4.13 Spatial Transcriptomics

The previously published human visium spatial transcriptomic (E‐MTAB‐10367) datasets were downloaded from ArrayExpress database and were visualized as tiled tiffs for analysis.

### 4.14 Isolation of Maxillofacial Derived Mesenchymal Stem Cells

Mandibles were harvested from the offspring of the recipient rats under anesthesia. Mmscs were flushed out from the mandibular condylar foramen, plated in cell culture dishes and cultured overnight. Then, nonadherent cells were removed by rinsing twice with phosphate‐buffered saline (PBS). The adherent cells were maintained in alpha minimum essential medium (α‐MEM) with 10% FBS, 2 mM Glutamax and 1% P/S at 37°C with 5% CO_2_.

### 4.15 In Vivo Implantation and Evaluation

All rats were anesthetized by inhaling 3%–4% isoflurane and maintained anesthetized by 1.5%–2% during subsequent surgery. After shaving and skin disinfection, the sciatic nerve was exposed along the posterior femoral space and transected to create a 15 mm sciatic nerve defect. Then the nerve scaffold (in the YE, OE, OG, OC, and OCG groups) or 180° rotated autograft nerve (in the OA group) was sutured to proximal and distal residual ends of the injured nerve using 8‐0 PLGA suture thread. Afterward, the outer muscle tissue and skin tissue were sutured sequentially with 5‐0 sutures thread and applied with ≈150 000 units of penicillin to prevent infection. The Zhejiang University Medical School Animal Care and Use Committee, and the Zhejiang Academy of Medical Sciences Animal Care and Use Committee approved all rat protocols and experiments (approval number ZJCLA‐IACUC‐20010227).

### 4.16 Live Imaging

The mixture of 0.3 mg Cy7‐N‐hydroxysuccinimide (Cy7‐NHS) ester, 2 mg Ga‐PTA nanoparticles and 2 mL DMF were stirred in dark at 25°C for 6 h. The Cy7 labeled Ga‐PTA nanoparticles were collected by ultracentrifugation and purified by ethanol/water (3:1, v/v) for three times, then vacuum dried for further use. Afterward, Cy7 labeled Ga‐PTA was injected into nerve conduits and deployed into the intermuscular space adjacent to the sciatic nerve. After surgery and 7 days after deploytion, rat live fluorescence imaging was carried out to observe the self‐disintegration and biodistribution of nanoparticles in vivo.

### 4.17 Neurological Function Evaluation

Thermal hyperalgesia in rat was evaluated using a hot/cold plate (RWD) to reflect the recovery of neurosensory function. The time of hind foot retraction was recorded for three times on both the normal side and the experimental side and the average value of each measurement was calculated.

The DAS assay was used to assess muscle refunctionalization and recovery of neuromotor function. Each rat was suspended rapidly and briefly in the air to elicit hindlimb extension and finger abduction. Rats with abnormal finger abduction responses or hind paw deformity were excluded from the study. Each rat's finger abduction response was scored using a 5‐point scale ranging from normal reflex (DAS 0) to complete inhibition of finger abduction reflex (DAS 4). Each rat repeated three times and obtained a mean value.

A grip strength meter system (RWD) was used to determine the hindlimb grip strength of the rat. The task exploits the tendency of the rats to grasp a horizontal metal rod while being pulled at the tail. Three trials over the course of 1 min were conducted for each mouse and the mean values were used to represent the grip strength of a mouse.

Paw volume was determined by water displacement plethysmometer (RWD), a microcontrolled volume meter specially designed for accurate measurement of the rodent paw swelling. The hind paws of rats were dipped into the water filled Perspex cell. A transducer of original design recorded small differences in water level, which indicated the exact volume of the paw.

Gait analysis was performed using the Noldus CatWalk XT system following the instructions of CatWalk XT 10.6 Reference Manual. Rats were placed at the far end of the CatWalk corridor and gait parameters recorded during each successful transverse of the track in either direction (set the maximum run duration for 20 s and the maximum run variation for 75%). A minimum of three successful runs were achieved for each rat. Rats unable to ambulate across the track were excluded from analysis. CatWalk XT software was used to analyze gait parameters and the following variables of both experimental (E) and normal (N) sides were simultaneously measured: the length (PL), width (TW), and middle toe width (MTW) of the toe map. The sciatic nerve function index (SFI, usually varies from −100 (total mutilation) to 0 (properly connected nerves)) was calculated using the following formula:

$$\begin{array}{cc} SFI= & -38.3\times \frac{PL^{E}-PL^{N}}{PL^{N}}+109.5\times \frac{TW^{E}-TW^{N}}{TW^{N}}\\ & +13.3\times \frac{ITW^{E}-ITW^{N}}{ITW^{N}} \end{array}$$

### 4.18 Histological Assessment

Sciatic nerve and gastrocnemius muscle specimens were fixed in 4% paraformaldehyde solution for staining and histological analysis. Nerve tissue was stained with HE and TB, while muscle tissue was stained with HE and Masson's trichromatic, and morphological correlation analysis was completed by Image J software. As for immunofluorescence and immunohistochemistry staining, nerve and muscle tissues were stained based on different protocols, and the images were captured by a fluorescence microscope and analyzed by Image J software.

### 4.19 Gastrocnemius Muscle Weightlessness

16 week post‐surgery, the gastrocnemius muscles on the both side of the rats were removed and weighed. Calculate the ratio of the weight of the gastrocnemius muscle on the surgical side to the normal side to evaluate reinnervation of the target muscle.

### 4.20 Electrophysiological Analysis

Before the final sampling, an electrophysiological evaluation was performed on the rats. After isoflurane inhalation anesthesia and sciatic nerve exposion, Braintech neurolego multichannel electrophysiological system was used to send a single stable electrical signal by placing bipolar electrodes at both ends of the regenerated nerve. The grounding electrode was placed in the superficial muscle layer and the receiving electrode was implanted into the gastrocnemius abdomen. The straight‐line distance between the two electrodes was 1.5 cm. The electromyography was recorded with an accuracy of 0.5 mm. The stimulus intensity was 10 mA, the frequency was 1 Hz, and the duration was 0.2 ms. The nerve conducting velocity (NCV), latency and amplitude of the compound muscle action potential (CMAP) were calculated. The nerve conduction velocity (NCV) was derived by dividing this distance by the onset latency. The compound muscle action potential (CMAP) was assessed via the latency period, peak‐to‐peak amplitude of the evoked potential waveform and NCV. These indexes were used to evaluate the number of nerve fibers in the sciatic nerve trunk that can effectively conduct nerve impulses.

### 4.21 Toxicity Testing

The heart, liver, spleen, lungs, and kidneys of rats were harvested in the 16th week after surgery. The corresponding sections were obtained and performed HE staining to detect the effects of different intervention.

### 4.22 Statistical Analysis

Statistical analysis and graphs were generated using R software V4.0.2, SPSS software V26.0, and GraphPad Prism 8.0. Student's *t*‐test was used to analyze two sets of data with normal distributions and homogeneous variances. One‐way analysis of variance (ANOVA) test was used to compare means among multigroup groups. Wilcoxon signed‐rank test was used for subgroup analysis. Two‐sided *p* <0.05 was used as the criterion for a statistically significant difference. All the experiments were performed with at least three replicates.

## Author Contributions

N.Z. performed all of the in‐vitro and animal experiments and wrote the paper with input from all the authors. L.T. performed the fabrication and characterization. L.P. performed the single‐cell analysis and data analysis. J.Z., L.L., X.Z., Y.L., and Z.L. assisted to perform the animal experiments and characterization. X.Y. assisted in the design of the nerve conduit. M.Y., Y.H., H.M., and M.S. organized the project and gave project suggestion.

## Conflicts of Interest

The authors declare no competing interests.

## Supporting information




**Supporting File**: advs77351‐sup‐0001‐SuppMat.docx.

## Data Availability

The data that support the findings of this study are available from the corresponding author upon reasonable request.
