# Supplementary material for: A Microenvironment‐Adaptive Scaffold Rejuvenates Peripheral Nerve Regeneration During Aging Via Dual Modulation of Ferroptotic Stimuli in Schwann Cells
Source: Adv Sci (Weinh). 2026 Aug 25:e77351. Online ahead of print. doi: 10.1002/advs.77351 (PMC13505465; doi:10.1002/advs.77351)
Supplement: Supplementary file 1 [file ADVS-9999-e77351-s001.docx]

Supplementary Materials for

**A microenvironment-adaptive scaffold rejuvenates peripheral nerveregeneration during aging via dual modulation of ferroptotic stimuli in Schwann cells**

Ning Zhan *et al.*

*Mengfei Yu, Ph.D.Email: yumengfei@zju.edu.cn

*Yong He, Ph.D.Email: [yongqin@zju.edu.cn](mailto:yongqin@zju.edu.cn)

**Extended Data Figure1.** (A) Hematoxylin-eosin (HE) staining of the regenerated nerves. Scale bar = 200 μm. (B) Toluidine blue (TB) staining of the regenerated nerves. Scale bar = 200 μm. (C) UMAP dimensionality reduction of the total cells. (D) Included single-cell datasets before data cleaning. (E) UMAP plot of the total cells labeled by groups. (F) Dot plot for expression levels of cell markers genes. (G) UMAP plot of cell types in different groups. (H) Proportion of cell types of schwann cells in different groups. (I) Violin plots for expression levels of top5 differential genes of different celltypes. (J) Gene ontology (GO) and Kyoto Encyclopedia of Genes and Genomes (KEGG) enrichment of the differentially expressed genes. (K) Violin plots for expression levels of the ferroptosis-related genes in single cell data. *p < 0.05, **p < 0.01, ***p < 0.001. Y, young control group; YI, young nerve injury group; O, aging control group; OI, aging nerve injury group.

**Extended Data Figure2.** (A) Histogram for the distribution of the size of Ga-PTA nanoparticles observed by SEM. (B) Zeta potential for hydrated PTA nanoparticles and hydrated Ga-PTA nanoparticles evaluated by dynamic light scattering (DLS). (C) The pattern of X-ray powder diffraction (XRD) of PTA, Ga-PTA nanoparticles and Gallium. (D) Live-death staining of RSC96 cells before and after intervention and its quantification data. Scale bar = 200 μm. (E) CCK-8 assays of RSC96 cells before and after intervention. (F) Wound healing experiments of RSC96 cells before and after intervention and its quantification data. (G) Quantitative RT-PCR analysis of MPZ and NGFR expression in RSC96 cells before and after intervention. *p < 0.05, **p < 0.01, ***p < 0.001. Y, young control group; O, aging control group; OG, aging Ga-PTA-intervention group. YS, Schwann cells; SS, senescent Schwann cell models; SSG, Ga-PTA-intervention senescent Schwann cell models.

**Extended Data Figure3.** (A) Immunohistochemistry staining for GPX4 of the regenerated nerves and its quantification data. Scale bar = 50 μm. (B) Immunofluorescence staining for Neun AND S100B of the regenerated nerves and its quantification data. Scale bar = 200 μm. (C) Senescence-associated beta-galactosidase (SA-β-gal) staining of RSC96 cells before and after intervention. Scale bar = 50 μm. (D) Violin plots for expression levels of SASP-related genes in single cell data of YI and OI group. *p < 0.05, **p < 0.01, ***p < 0.001. YE, young hollow conduit group; OE, aging hollow conduit group; OG, aging Ga-PTA-intervention group; YI, young nerve injury group; OI, aging nerve injury group. YS, Schwann cells; SS, senescent Schwann cell models; SSG, Ga-PTA-intervention senescent Schwann cell models.

**Extended Data Figure4.** (A) Pseudotime trajectory analysis of of Schwann cells in the aged group. (B) Pseudotime kinetics of the SASP-related genes from the root of the trajectory to the end of the trajectory. (C) Projection of the key node genes in the SASP signaling network on the trajectory. (D) Protein–protein interaction (PPI) network of the SASP-related genes. (E) Spatial mapping of gene expression for the key node genes in the SASP signaling network in different stage of the developing limb. (F) Spatial mapping of gene expression for the ferroptosis-related genes in different stage of the developing limb. (G) Spatial mapping of gene expression for the marker genes of mSC and dSC in different stage of the developing limb.

**Extended Data Figure5.** (A) Overview of the analysis pipeline to integrate human and rat scRNA-seq data. (B) Proportion of cell types in different samples. (C) UMAP plot of cell types in total cells. (D) UMAP plot of the total cells labeled by species. (E) PCA plot of the total cells labeled by species. (F) PCA plot of the total cells labeled by samples.

**Extended Data Figure6.** (A) Live-death staining of Mmscs treated by different dose of Ga-PTA. Scale bar = 200 μm. (B) Live-death staining of Bmscs treated by different dose of Ga-PTA. Scale bar = 200 μm. (C) The immunofluorescence images of S100B (green) and actin cytoskeleton (red) in Bmscs after Ga-PTA intervention and its quantification data. Scale bar = 50 μm. (D) Senescence-associated beta-galactosidase (SA-β-gal) staining of Mmscs before and after intervention. Scale bar =250 μm. *p < 0.05, **p < 0.01, ***p < 0.001. B, Bmscs; BG, Ga-PTA intervened Bmscs; Y, young control group; O, aging control group; OG, aging Ga-PTA-intervention group. M, Mmscs; SM, senescent Mmsc models; SMG, Ga-PTA-intervention senescent Mmsc models.

**Extended Data Figure7.** (A) Live-death staining of the senescent PC12s before and after intervention and its quantification data. Scale bar = 200 μm. (B) Wound healing experiments of the senescent PC12s before and after intervention and its quantification data. *p < 0.05, **p < 0.01, ***p < 0.001. O, aging control group; OG, aging Ga-PTA-intervention group. SN, senescent PC12 cell models; SNG, Ga-PTA-intervention senescent PC12 cell models.

**Extended Data Figure8.** (A) Live-death staining of MMSCs cultured in hydrogel with different ratio of Matrigel: GelMA. Scale bar = 200 μm. (B) CCK-8 assays of MMSCs cultured in hydrogel with different ratio of Matrigel: GelMA. (C-F) The radial and axial stiffness, maximum stress and maximum load of empty conduits and the conduits filled with Ga-PTA nanoparticles and MMSC hydrogel. *p < 0.05, **p < 0.01, ***p < 0.001.

**Extended Data Figure9.** (A) Hematoxylin-eosin (HE) staining of the regenerated nerves at 16 weeks post-operation. Scale bar = 200 μm. (B) Toluidine blue (TB) staining of the regenerated nerves at 16 weeks post-operation and its quantification data. Scale bar = 200 μm. (C) Immunofluorescence images of cross-sectional gastrocnemius muscle on the operated side for Myosin heavy chain (MYH). Quantitative analysis of intensity is also shown. Scale bar = 200μm. *p < 0.05, **p < 0.01, ***p < 0.001. OS, aging sham group; OA, aging autologous nerve anastomosis group; OC, aging PNI group treated with Mmsc-loaded nerve conduits; OCG, aging PNI group treated with Ga-PTA and Mmsc-loaded nerve conduits.

**Extended Data Figure10.** (A-E) Hematoxylin-eosin (HE) staining of the lung,liver, spleen,kidney and heart at 16 weeks post-operation. Scale bar = 200 μm. *p < 0.05, **p < 0.01, ***p < 0.001. OS, aging sham group; OA, aging autologous nerve anastomosis group; OC, aging PNI group treated with Mmsc-loaded nerve conduits; OCG, aging PNI group treated with Ga-PTA and Mmsc-loaded nerve conduits.

Extended Data Table 1. Results of Routine Blood Test.

| Routine Blood Test of OS-1 | | | | |
| --- | --- | --- | --- | --- |
| Parameter |  | Result | Reference Range | Unit |
| White Blood Cell Count | WBC | 1.35 | 0.8-6.8 | 10^9/L |
| Red Blood Cell Count | RBC | 1.14 | 6.36-9.42 | 10^12/L |
| Hemoglobin | HGB | 25 | 110-143 | g/L |
| Mean Corpuscular Volume | MCV | 56.7 | 48.2-58.3 | fL |
| Platelet Count | PLT | 94 | 450-1590 | 10^9/L |
| Lymphocyte Count | Lym# | 1.25 | 0.7-5.7 | 10^9/L |
| Mid-cell Count | Mid# | 0.04 | 0-0.3 | 10^9/L |
| Granulocyte Count | GR# | 0.06 | 0.1-1.8 | 10^9/L |
| Lymphocyte Percentage | Lym% | 92.9 | 55.8-90.6 | % |
| Mid-cell Percentage | Mid% | 2.7 | 1.8-6 | % |
| Granulocyte Percentage | GR% | 4.4 | 8.6-38.9 | % |
| Platelet-Large Cell Ratio | P_LCR | 19.2 | 13-50 | % |
| Platelet-Large Cell Count | P_LCC | 18 | 10-100 | 10^9/L |
| Hematocrit | HCT | 6.4 | 34.6-44.6 | % |
| Mean Corpuscular Hemoglobin Concentration | MCHC | 391 | 302-353 | g/L |
| Red Cell Distribution Width-Coefficient of Variation | RDW_CV | 8.9 | 11-17 | % |
| Red Cell Distribution Width-Standard Deviation | RDW_SD | 51.0 | 33-50 | fL |
| Mean Corpuscular Hemoglobin | MCH | 21.9 | 15.8-19 | pg |
| Mean Platelet Volume | MPV | 9.7 | 3.8-6 | fL |
| Plateletcrit | PCT | 0.091 | 0.15-0.3 | % |
| Platelet Distribution Width | PDW | 22.7 | 15-17 | % |

| Routine Blood Test of OS-2 | | | | |
| --- | --- | --- | --- | --- |
| Parameter |  | Result | Reference Range | Unit |
| White Blood Cell Count | WBC | 3.25 | 0.8-6.8 | 10^9/L |
| Red Blood Cell Count | RBC | 6.17 | 6.36-9.42 | 10^12/L |
| Hemoglobin | HGB | 115 | 110-143 | g/L |
| Mean Corpuscular Volume | MCV | 53.9 | 48.2-58.3 | fL |
| Platelet Count | PLT | 360 | 450-1590 | 10^9/L |
| Lymphocyte Count | Lym# | 2.14 | 0.7-5.7 | 10^9/L |
| Mid-cell Count | Mid# | 0.14 | 0-0.3 | 10^9/L |
| Granulocyte Count | GR# | 0.97 | 0.1-1.8 | 10^9/L |
| Lymphocyte Percentage | Lym% | 66.0 | 55.8-90.6 | % |
| Mid-cell Percentage | Mid% | 4.3 | 1.8-6 | % |
| Granulocyte Percentage | GR% | 29.7 | 8.6-38.9 | % |
| Platelet-Large Cell Ratio | P_LCR | 21.1 | 13-50 | % |
| Platelet-Large Cell Count | P_LCC | 76 | 10-100 | 10^9/L |
| Hematocrit | HCT | 33.3 | 34.6-44.6 | % |
| Mean Corpuscular Hemoglobin Concentration | MCHC | 345 | 302-353 | g/L |
| Red Cell Distribution Width-Coefficient of Variation | RDW_CV | 10.2 | 11-17 | % |
| Red Cell Distribution Width-Standard Deviation | RDW_SD | 60.5 | 33-50 | fL |
| Mean Corpuscular Hemoglobin | MCH | 18.6 | 15.8-19 | pg |
| Mean Platelet Volume | MPV | 9.6 | 3.8-6 | fL |
| Plateletcrit | PCT | 0.346 | 0.15-0.3 | % |
| Platelet Distribution Width | PDW | 20.6 | 15-17 | % |

| Routine Blood Test of OS-3 | | | | |
| --- | --- | --- | --- | --- |
| Parameter |  | Result | Reference Range | Unit |
| White Blood Cell Count | WBC | 2.42 | 0.8-6.8 | 10^9/L |
| Red Blood Cell Count | RBC | 5.97 | 6.36-9.42 | 10^12/L |
| Hemoglobin | HGB | 116 | 110-143 | g/L |
| Mean Corpuscular Volume | MCV | 54.5 | 48.2-58.3 | fL |
| Platelet Count | PLT | 338 | 450-1590 | 10^9/L |
| Lymphocyte Count | Lym# | 1.31 | 0.7-5.7 | 10^9/L |
| Mid-cell Count | Mid# | 0.11 | 0-0.3 | 10^9/L |
| Granulocyte Count | GR# | 1.00 | 0.1-1.8 | 10^9/L |
| Lymphocyte Percentage | Lym% | 54.2 | 55.8-90.6 | % |
| Mid-cell Percentage | Mid% | 4.7 | 1.8-6 | % |
| Granulocyte Percentage | GR% | 41.1 | 8.6-38.9 | % |
| Platelet-Large Cell Ratio | P_LCR | 19.8 | 13-50 | % |
| Platelet-Large Cell Count | P_LCC | 67 | 10-100 | 10^9/L |
| Hematocrit | HCT | 32.6 | 34.6-44.6 | % |
| Mean Corpuscular Hemoglobin Concentration | MCHC | 356 | 302-353 | g/L |
| Red Cell Distribution Width-Coefficient of Variation | RDW_CV | 10.2 | 11-17 | % |
| Red Cell Distribution Width-Standard Deviation | RDW_SD | 59.6 | 33-50 | fL |
| Mean Corpuscular Hemoglobin | MCH | 19.4 | 15.8-19 | pg |
| Mean Platelet Volume | MPV | 9.4 | 3.8-6 | fL |
| Plateletcrit | PCT | 0.318 | 0.15-0.3 | % |
| Platelet Distribution Width | PDW | 19.8 | 15-17 | % |

| Routine Blood Test of OA-1 | | | | |
| --- | --- | --- | --- | --- |
| Parameter |  | Result | Reference Range | Unit |
| White Blood Cell Count | WBC | 0.45 | 0.8-6.8 | 10^9/L |
| Red Blood Cell Count | RBC | 2.72 | 6.36-9.42 | 10^12/L |
| Hemoglobin | HGB | 58 | 110-143 | g/L |
| Mean Corpuscular Volume | MCV | 53.6 | 48.2-58.3 | fL |
| Platelet Count | PLT | 150 | 450-1590 | 10^9/L |
| Lymphocyte Count | Lym# | 0.44 | 0.7-5.7 | 10^9/L |
| Mid-cell Count | Mid# | 0.01 | 0-0.3 | 10^9/L |
| Granulocyte Count | GR# | 0.01 | 0.1-1.8 | 10^9/L |
| Lymphocyte Percentage | Lym% | 97.4 | 55.8-90.6 | % |
| Mid-cell Percentage | Mid% | 2.59 | 1.8-6 | % |
| Granulocyte Percentage | GR% | 0.01 | 8.6-38.9 | % |
| Platelet-Large Cell Ratio | P_LCR | 23.4 | 13-50 | % |
| Platelet-Large Cell Count | P_LCC | 35 | 10-100 | 10^9/L |
| Hematocrit | HCT | 14.6 | 34.6-44.6 | % |
| Mean Corpuscular Hemoglobin Concentration | MCHC | 397 | 302-353 | g/L |
| Red Cell Distribution Width-Coefficient of Variation | RDW_CV | 10.9 | 11-17 | % |
| Red Cell Distribution Width-Standard Deviation | RDW_SD | 65.0 | 33-50 | fL |
| Mean Corpuscular Hemoglobin | MCH | 21.3 | 15.8-19 | pg |
| Mean Platelet Volume | MPV | 9.5 | 3.8-6 | fL |
| Plateletcrit | PCT | 0.143 | 0.15-0.3 | % |
| Platelet Distribution Width | PDW | 20.5 | 15-17 | % |

| Routine Blood Test of OA-2 | | | | |
| --- | --- | --- | --- | --- |
| Parameter |  | Result | Reference Range | Unit |
| White Blood Cell Count | WBC | 5.02 | 0.8-6.8 | 10^9/L |
| Red Blood Cell Count | RBC | 6.01 | 6.36-9.42 | 10^12/L |
| Hemoglobin | HGB | 124 | 110-143 | g/L |
| Mean Corpuscular Volume | MCV | 56.8 | 48.2-58.3 | fL |
| Platelet Count | PLT | 385 | 450-1590 | 10^9/L |
| Lymphocyte Count | Lym# | 2.24 | 0.7-5.7 | 10^9/L |
| Mid-cell Count | Mid# | 0.32 | 0-0.3 | 10^9/L |
| Granulocyte Count | GR# | 2.46 | 0.1-1.8 | 10^9/L |
| Lymphocyte Percentage | Lym% | 44.7 | 55.8-90.6 | % |
| Mid-cell Percentage | Mid% | 6.3 | 1.8-6 | % |
| Granulocyte Percentage | GR% | 49.0 | 8.6-38.9 | % |
| Platelet-Large Cell Ratio | P_LCR | 40.3 | 13-50 | % |
| Platelet-Large Cell Count | P_LCC | 155 | 10-100 | 10^9/L |
| Hematocrit | HCT | 34.2 | 34.6-44.6 | % |
| Mean Corpuscular Hemoglobin Concentration | MCHC | 363 | 302-353 | g/L |
| Red Cell Distribution Width-Coefficient of Variation | RDW_CV | 11.9 | 11-17 | % |
| Red Cell Distribution Width-Standard Deviation | RDW_SD | 58.2 | 33-50 | fL |
| Mean Corpuscular Hemoglobin | MCH | 20.6 | 15.8-19 | pg |
| Mean Platelet Volume | MPV | 9.9 | 3.8-6 | fL |
| Plateletcrit | PCT | 0.381 | 0.15-0.3 | % |
| Platelet Distribution Width | PDW | 20.2 | 15-17 | % |

| Routine Blood Test of OA-3 | | | | |
| --- | --- | --- | --- | --- |
| Parameter |  | Result | Reference Range | Unit |
| White Blood Cell Count | WBC | 0.69 | 0.8-6.8 | 10^9/L |
| Red Blood Cell Count | RBC | 2.72 | 6.36-9.42 | 10^12/L |
| Hemoglobin | HGB | 56 | 110-143 | g/L |
| Mean Corpuscular Volume | MCV | 51.8 | 48.2-58.3 | fL |
| Platelet Count | PLT | 134 | 450-1590 | 10^9/L |
| Lymphocyte Count | Lym# | 0.65 | 0.7-5.7 | 10^9/L |
| Mid-cell Count | Mid# | 0.02 | 0-0.3 | 10^9/L |
| Granulocyte Count | GR# | 0.02 | 0.1-1.8 | 10^9/L |
| Lymphocyte Percentage | Lym% | 94.3 | 55.8-90.6 | % |
| Mid-cell Percentage | Mid% | 2.7 | 1.8-6 | % |
| Granulocyte Percentage | GR% | 3.0 | 8.6-38.9 | % |
| Platelet-Large Cell Ratio | P_LCR | 31.8 | 13-50 | % |
| Platelet-Large Cell Count | P_LCC | 43 | 10-100 | 10^9/L |
| Hematocrit | HCT | 14.1 | 34.6-44.6 | % |
| Mean Corpuscular Hemoglobin Concentration | MCHC | 397 | 302-353 | g/L |
| Red Cell Distribution Width-Coefficient of Variation | RDW_CV | 9.3 | 11-17 | % |
| Red Cell Distribution Width-Standard Deviation | RDW_SD | 53.2 | 33-50 | fL |
| Mean Corpuscular Hemoglobin | MCH | 20.6 | 15.8-19 | pg |
| Mean Platelet Volume | MPV | 9.4 | 3.8-6 | fL |
| Plateletcrit | PCT | 0.126 | 0.15-0.3 | % |
| Platelet Distribution Width | PDW | 20.6 | 15-17 | % |

| Routine Blood Test of OC-1 | | | | |
| --- | --- | --- | --- | --- |
| Parameter |  | Result | Reference Range | Unit |
| White Blood Cell Count | WBC | 0.96 | 0.8-6.8 | 10^9/L |
| Red Blood Cell Count | RBC | 4.17 | 6.36-9.42 | 10^12/L |
| Hemoglobin | HGB | 86 | 110-143 | g/L |
| Mean Corpuscular Volume | MCV | 53.6 | 48.2-58.3 | fL |
| Platelet Count | PLT | 250 | 450-1590 | 10^9/L |
| Lymphocyte Count | Lym# | 0.91 | 0.7-5.7 | 10^9/L |
| Mid-cell Count | Mid# | 0.02 | 0-0.3 | 10^9/L |
| Granulocyte Count | GR# | 0.03 | 0.1-1.8 | 10^9/L |
| Lymphocyte Percentage | Lym% | 95.2 | 55.8-90.6 | % |
| Mid-cell Percentage | Mid% | 1.9 | 1.8-6 | % |
| Granulocyte Percentage | GR% | 2.9 | 8.6-38.9 | % |
| Platelet-Large Cell Ratio | P_LCR | 30.4 | 13-50 | % |
| Platelet-Large Cell Count | P_LCC | 76 | 10-100 | 10^9/L |
| Hematocrit | HCT | 22.4 | 34.6-44.6 | % |
| Mean Corpuscular Hemoglobin Concentration | MCHC | 384 | 302-353 | g/L |
| Red Cell Distribution Width-Coefficient of Variation | RDW_CV | 9.4 | 11-17 | % |
| Red Cell Distribution Width-Standard Deviation | RDW_SD | 54.6 | 33-50 | fL |
| Mean Corpuscular Hemoglobin | MCH | 20.6 | 15.8-19 | pg |
| Mean Platelet Volume | MPV | 9.3 | 3.8-6 | fL |
| Plateletcrit | PCT | 0.233 | 0.15-0.3 | % |
| Platelet Distribution Width | PDW | 20.0 | 15-17 | % |

| Routine Blood Test of OC-2 | | | | |
| --- | --- | --- | --- | --- |
| Parameter |  | Result | Reference Range | Unit |
| White Blood Cell Count | WBC | 1.30 | 0.8-6.8 | 10^9/L |
| Red Blood Cell Count | RBC | 4.25 | 6.36-9.42 | 10^12/L |
| Hemoglobin | HGB | 83 | 110-143 | g/L |
| Mean Corpuscular Volume | MCV | 50.9 | 48.2-58.3 | fL |
| Platelet Count | PLT | 248 | 450-1590 | 10^9/L |
| Lymphocyte Count | Lym# | 1.05 | 0.7-5.7 | 10^9/L |
| Mid-cell Count | Mid# | 0.05 | 0-0.3 | 10^9/L |
| Granulocyte Count | GR# | 0.20 | 0.1-1.8 | 10^9/L |
| Lymphocyte Percentage | Lym% | 81.1 | 55.8-90.6 | % |
| Mid-cell Percentage | Mid% | 4.1 | 1.8-6 | % |
| Granulocyte Percentage | GR% | 14.8 | 8.6-38.9 | % |
| Platelet-Large Cell Ratio | P_LCR | 16.9 | 13-50 | % |
| Platelet-Large Cell Count | P_LCC | 42 | 10-100 | 10^9/L |
| Hematocrit | HCT | 21.6 | 34.6-44.6 | % |
| Mean Corpuscular Hemoglobin Concentration | MCHC | 384 | 302-353 | g/L |
| Red Cell Distribution Width-Coefficient of Variation | RDW_CV | 10.9 | 11-17 | % |
| Red Cell Distribution Width-Standard Deviation | RDW_SD | 59.4 | 33-50 | fL |
| Mean Corpuscular Hemoglobin | MCH | 19.5 | 15.8-19 | pg |
| Mean Platelet Volume | MPV | 9.1 | 3.8-6 | fL |
| Plateletcrit | PCT | 0.226 | 0.15-0.3 | % |
| Platelet Distribution Width | PDW | 20.0 | 15-17 | % |

| Routine Blood Test of OC-3 | | | | |
| --- | --- | --- | --- | --- |
| Parameter |  | Result | Reference Range | Unit |
| White Blood Cell Count | WBC | 0.91 | 0.8-6.8 | 10^9/L |
| Red Blood Cell Count | RBC | 4.30 | 6.36-9.42 | 10^12/L |
| Hemoglobin | HGB | 97 | 110-143 | g/L |
| Mean Corpuscular Volume | MCV | 55.9 | 48.2-58.3 | fL |
| Platelet Count | PLT | 215 | 450-1590 | 10^9/L |
| Lymphocyte Count | Lym# | 0.87 | 0.7-5.7 | 10^9/L |
| Mid-cell Count | Mid# | 0.02 | 0-0.3 | 10^9/L |
| Granulocyte Count | GR# | 0.02 | 0.1-1.8 | 10^9/L |
| Lymphocyte Percentage | Lym% | 95.9 | 55.8-90.6 | % |
| Mid-cell Percentage | Mid% | 1.9 | 1.8-6 | % |
| Granulocyte Percentage | GR% | 2.2 | 8.6-38.9 | % |
| Platelet-Large Cell Ratio | P_LCR | 21.2 | 13-50 | % |
| Platelet-Large Cell Count | P_LCC | 46 | 10-100 | 10^9/L |
| Hematocrit | HCT | 24.0 | 34.6-44.6 | % |
| Mean Corpuscular Hemoglobin Concentration | MCHC | 404 | 302-353 | g/L |
| Red Cell Distribution Width-Coefficient of Variation | RDW_CV | 11.0 | 11-17 | % |
| Red Cell Distribution Width-Standard Deviation | RDW_SD | 58.5 | 33-50 | fL |
| Mean Corpuscular Hemoglobin | MCH | 22.6 | 15.8-19 | pg |
| Mean Platelet Volume | MPV | 9.6 | 3.8-6 | fL |
| Plateletcrit | PCT | 0.206 | 0.15-0.3 | % |
| Platelet Distribution Width | PDW | 19.8 | 15-17 | % |

| Routine Blood Test of OCG-1 | | | | |
| --- | --- | --- | --- | --- |
| Parameter |  | Result | Reference Range | Unit |
| White Blood Cell Count | WBC | 4.42 | 0.8-6.8 | 10^9/L |
| Red Blood Cell Count | RBC | 4.00 | 6.36-9.42 | 10^12/L |
| Hemoglobin | HGB | 86 | 110-143 | g/L |
| Mean Corpuscular Volume | MCV | 55.9 | 48.2-58.3 | fL |
| Platelet Count | PLT | 341 | 450-1590 | 10^9/L |
| Lymphocyte Count | Lym# | 2.84 | 0.7-5.7 | 10^9/L |
| Mid-cell Count | Mid# | 0.30 | 0-0.3 | 10^9/L |
| Granulocyte Count | GR# | 1.28 | 0.1-1.8 | 10^9/L |
| Lymphocyte Percentage | Lym% | 64.2 | 55.8-90.6 | % |
| Mid-cell Percentage | Mid% | 6.7 | 1.8-6 | % |
| Granulocyte Percentage | GR% | 29.1 | 8.6-38.9 | % |
| Platelet-Large Cell Ratio | P_LCR | 31.2 | 13-50 | % |
| Platelet-Large Cell Count | P_LCC | 106 | 10-100 | 10^9/L |
| Hematocrit | HCT | 22.4 | 34.6-44.6 | % |
| Mean Corpuscular Hemoglobin Concentration | MCHC | 384 | 302-353 | g/L |
| Red Cell Distribution Width-Coefficient of Variation | RDW_CV | 10.6 | 11-17 | % |
| Red Cell Distribution Width-Standard Deviation | RDW_SD | 57.4 | 33-50 | fL |
| Mean Corpuscular Hemoglobin | MCH | 21.5 | 15.8-19 | pg |
| Mean Platelet Volume | MPV | 9.7 | 3.8-6 | fL |
| Plateletcrit | PCT | 0.331 | 0.15-0.3 | % |
| Platelet Distribution Width | PDW | 21.1 | 15-17 | % |

| Routine Blood Test of OCG-2 | | | | |
| --- | --- | --- | --- | --- |
| Parameter |  | Result | Reference Range | Unit |
| White Blood Cell Count | WBC | 1.69 | 0.8-6.8 | 10^9/L |
| Red Blood Cell Count | RBC | 4.93 | 6.36-9.42 | 10^12/L |
| Hemoglobin | HGB | 104 | 110-143 | g/L |
| Mean Corpuscular Volume | MCV | 55.9 | 48.2-58.3 | fL |
| Platelet Count | PLT | 422 | 450-1590 | 10^9/L |
| Lymphocyte Count | Lym# | 1.46 | 0.7-5.7 | 10^9/L |
| Mid-cell Count | Mid# | 0.07 | 0-0.3 | 10^9/L |
| Granulocyte Count | GR# | 0.16 | 0.1-1.8 | 10^9/L |
| Lymphocyte Percentage | Lym% | 86.6 | 55.8-90.6 | % |
| Mid-cell Percentage | Mid% | 4.2 | 1.8-6 | % |
| Granulocyte Percentage | GR% | 9.2 | 8.6-38.9 | % |
| Platelet-Large Cell Ratio | P_LCR | 21.5 | 13-50 | % |
| Platelet-Large Cell Count | P_LCC | 91 | 10-100 | 10^9/L |
| Hematocrit | HCT | 27.5 | 34.6-44.6 | % |
| Mean Corpuscular Hemoglobin Concentration | MCHC | 378 | 302-353 | g/L |
| Red Cell Distribution Width-Coefficient of Variation | RDW_CV | 11.3 | 11-17 | % |
| Red Cell Distribution Width-Standard Deviation | RDW_SD | 59.4 | 33-50 | fL |
| Mean Corpuscular Hemoglobin | MCH | 21.1 | 15.8-19 | pg |
| Mean Platelet Volume | MPV | 9.7 | 3.8-6 | fL |
| Plateletcrit | PCT | 0.409 | 0.15-0.3 | % |
| Platelet Distribution Width | PDW | 20.6 | 15-17 | % |

| Routine Blood Test of OCG-3 | | | | |
| --- | --- | --- | --- | --- |
| Parameter |  | Result | Reference Range | Unit |
| White Blood Cell Count | WBC | 2.88 | 0.8-6.8 | 10^9/L |
| Red Blood Cell Count | RBC | 4.34 | 6.36-9.42 | 10^12/L |
| Hemoglobin | HGB | 109 | 110-143 | g/L |
| Mean Corpuscular Volume | MCV | 61.6 | 48.2-58.3 | fL |
| Platelet Count | PLT | 447 | 450-1590 | 10^9/L |
| Lymphocyte Count | Lym# | 2.41 | 0.7-5.7 | 10^9/L |
| Mid-cell Count | Mid# | 0.12 | 0-0.3 | 10^9/L |
| Granulocyte Count | GR# | 0.35 | 0.1-1.8 | 10^9/L |
| Lymphocyte Percentage | Lym% | 83.7 | 55.8-90.6 | % |
| Mid-cell Percentage | Mid% | 4.1 | 1.8-6 | % |
| Granulocyte Percentage | GR% | 12.2 | 8.6-38.9 | % |
| Platelet-Large Cell Ratio | P_LCR | 29.1 | 13-50 | % |
| Platelet-Large Cell Count | P_LCC | 130 | 10-100 | 10^9/L |
| Hematocrit | HCT | 26.7 | 34.6-44.6 | % |
| Mean Corpuscular Hemoglobin Concentration | MCHC | 408 | 302-353 | g/L |
| Red Cell Distribution Width-Coefficient of Variation | RDW_CV | 11.2 | 11-17 | % |
| Red Cell Distribution Width-Standard Deviation | RDW_SD | 64.4 | 33-50 | fL |
| Mean Corpuscular Hemoglobin | MCH | 25.1 | 15.8-19 | pg |
| Mean Platelet Volume | MPV | 10.0 | 3.8-6 | fL |
| Plateletcrit | PCT | 0.447 | 0.15-0.3 | % |
| Platelet Distribution Width | PDW | 20.7 | 15-17 | % |

Extended Data Table 2. Primer sequences used for RT-qPCR.

| **Target genes (rat)** | **Primer sequences (5’-3’)** |
| --- | --- |
| Slc39a8 | F:TGCTTTCGGCATTTTGGTGG |
|  | R:CATCCCTGCGTTCTGGATCA |
| Slc7a1 | F:ACGAGCTGATAGGCAAACCC |
|  | R:ATGAACCCTCCCTCACCGTA |
| Tp53i11 | F:CGGTCAAAGATCAGCCAGGT |
|  | R:TAACAAAGAGGAGGCGGCTG |
| Tp53inp2 | F:CAGCTATACAGCTCCACCCG |
|  | R:GTGCTGCCGGTGACATAAAC |
| MPZ | F:GTGGTTTACACGGACAGGGAA |
|  | R:CCTTGGCATAGTGGAAGATTGA |
| NGFR | F:TGGAGCCGAAGGGGAGC |
|  | R:GTCTCCCTCTGGGACATTGC |
| GAPDH | F:AATGGGCAGCCGTTAGGAAA |
|  | R:GCGCCCAATACGACCAAATC |
